# “You never know what’s in front of you”: A mixed methods study of barriers and facilitators to physical activity among blind and low-vision adults with type 2 diabetes

**DOI:** 10.1371/journal.pone.0332565

**Published:** 2026-06-17

**Authors:** Emily J. Nicklett, Meredith L. Stensland, Weidi Qin, Tiffany Walker, Turnip Van Dyke, Deven Murray, Beatriz Morales-Hernandez, Giselle Reinhardt Gillis, Julia J. Crowley

**Affiliations:** 1 Department of Social Work, College for Health, Community and Policy, University of Texas at San Antonio, San Antonio, Texas, United States of America; 2 Department of Psychiatry & Behavioral Sciences, UT Health San Antonio, San Antonio, Texas, United States of America; 3 Sandra Rosenbaum School of Social Work, University of Wisconsin-Madison, Madison, Wisconsin, United States of America; 4 Vibrant Works, San Antonio, Texas, United States of America; Touro University California College of Pharmacy, UNITED STATES OF AMERICA

## Abstract

Individuals living with type 2 diabetes and vision loss experience unique, under-researched barriers to physical activity, despite the important role exercise plays in diabetes management. This study examined physical activity participation among blind and low-vision adults with type 2 diabetes (*n* = 30). Participants, ages 44–83, resided in Bexar County, Texas. Closed-ended surveys (conducted in English or Spanish) were administered to participants, followed by in-depth, semi-structured interviews. Interviews were audio-recorded, transcribed verbatim, and double-coded. Participants’ physical activity levels were determined using the Exercise Behaviors Scale. Data were analyzed using a convergent inductive-deductive design. Consistent with the Social-Ecological Model, five main themes and 11 subthemes emerged, depicting how individual/intrapersonal, interpersonal, institutional/organizational, community, and policy-level factors influenced participants’ physical activity participation. These factors can be specifically targeted both at local and broader levels to promote physical activity among individuals with concurrent type 2 diabetes and blindness/vision loss.

## Introduction

The Centers for Disease Control and Prevention [1] estimates that 14.7% of US adults, 38.1 million, have type 2 diabetes (T2D). Of those adults with T2D, an estimated 26.43%—9.6 million—have diabetic retinopathy (DR) [2], a leading cause of vision loss and blindness globally [3,4]. Nearly 1.8 million Americans have vision-threatening DR, making it a major contributor to blindness among working-age adults [2]. Regionally, Bexar County, Texas—where this study was conducted—faces an even greater burden. Approximately 15% of adults in Bexar County (≈239,000 people) have been diagnosed with T2D, exceeding the national average [5,6]. Among these individuals, about 28% exhibit signs of DR, mirroring national trends while highlighting a critical local public health concern [7]. These figures underscore the significant impact of vision loss due to diabetes both nationally and regionally, particularly in communities with high diabetes prevalence and socioeconomic inequalities [8,9].

Regular physical activity (PA) can slow the progression of diabetes-related complications, including vision-threatening retinopathy [10,11]. Although individuals with DR must take precautions during exercise to prevent falls and further retinal damage, PA is critical for preserving remaining vision, balance, functional capacity, and independence [12,13]. The American Diabetes Association recommends that people with T2D engage in at least 150 minutes per week of moderate-intensity aerobic PA, plus resistance training 2–3 times weekly [10]. However, only 20–40% of individuals with T2D meet these PA recommendations [1,14]. Prior research on this topic, though limited, suggests that PA participation is even lower among populations with diabetic retinopathy and associated vision loss [15–17]. For example, a study by Janevic et al. [15] using 2003 Diabetes Supplement data from the U.S. Health and Retirement Study (n = 1,811) found that participants with diabetic retinopathy were 44% less likely to meet PA guidelines compared with their diabetic counterparts without retinopathy. Another study by Loprinzi et al. [16] using accelerometer data from the 2003–2006 NHANES cycle (n = 770) found that participants with diabetes and impaired vision were significantly less likely than their sighted counterparts with diabetes to engage in moderate-to-vigorous (82%) and light-intensity PA (38%). Studies that assess PA in blind and low vision populations (not specific to T2D) report similar findings: Flynn et al. [18] examined PA levels among 310 self-identifying blind and vision-impaired adults in Ireland, showing that only 21.7% of participants met PA guidelines.

Individuals with T2D and additional comorbidities encounter significant barriers to engaging in PA as part of their diabetes regimen [19–21]. However, the unique challenges faced by individuals with both T2D and vision loss remain underexplored. Although prior research suggests that blindness and vision impairment can limit opportunities for PA [13], no studies to date have specifically examined how these dual conditions interact to affect PA engagement within diabetes care. While not specific to PA, an emerging body of qualitative research has begun to explore the lived experiences of individuals with T2D and vision loss [22–24]. For example, Devenney & O’Neill [22] found that individuals with vision loss due to diabetic retinopathy expressed a heightened need for support in managing their diabetes, citing loss of independence and mobility as key barriers. Kaminsky et al. [23] emphasized the role of physical and social environments, as well as psychological adaptation, in shaping rehabilitation outcomes for individuals with diabetic retinopathy. Similarly, Williams [24] identified barriers and facilitators to diabetes self-management among adults with visual impairment and diabetes. While these studies offer valuable insights into the broader lived experience of vision loss in the context of diabetes, they do not address how these experiences specifically influence PA engagement. This study seeks to fill that gap by examining the intersection of T2D, vision loss, and PA within diabetes care.

Based on the Social-Ecological Model [25], experiences of vision loss and T2D unfold across intrapersonal, interpersonal, organizational, community, and public policy levels. Intrapersonal factors can be physical—such as the additional challenges imposed by the loss of independence and comorbid complications [22,23]—or psychosocial, such as motivation [18] or the need for psychological adaptation and acceptance of vision loss for health behavior change [22,23,26]. Additionally, prior research highlights interpersonal and social/structural factors affecting diabetes self-management in adults with diabetes and vision loss, including social/peer support [22,23], accessible equipment [24], individualized diabetes education [24], transportation [18], and built environment barriers [23].

Prior studies on self-management among adults with T2D and vision loss have identified various barriers and facilitators [22–24]. However, these studies have not specifically explored PA behaviors and experiences. As a result, there remains a critical gap in understanding both the factors that hinder PA participation and those that promote engagement in this population. Second, our limited understanding of PA participation among adults with T2D and vision loss has been derived predominantly from separate and distinct quantitative or qualitative studies; prior work has either characterized whether there were differences in PA levels (quantitative studies) or examined narratives of diabetes self-management (qualitative studies). A mixed methods approach offers the ability to capture both measurable activity patterns and the lived experiences underlying them [27,28], with key insights provided by participant lived experience and engagement of community partners [29,30]. The present study addresses critical gaps in the literature on factors that hinder and facilitate participation in PA among those with T2D and vision loss, integrating quantitative data on PA predictors and levels with qualitative insights from adults with T2D and vision loss.

## Methods

This study was conducted in Bexar County, Texas (including the city of San Antonio) from January 2023 to October 2024 in partnership with Vibrant Works, a nonprofit community organization providing employment, education, independent living, and rehabilitation services to people who are blind and vision impaired, as well as their caregivers. Collaborating with Vibrant Works—throughout the conception, design, data collection, and analysis—grounded the study and facilitated recruitment of a hard-to-reach population. The study aims to (1) understand the challenges and strategies that support PA participation among individuals living with T2D and vision loss; and (2) inform current health promotion programs and services for blind and low-vision adults with T2D.

### Participant recruitment

**Fig 1:**
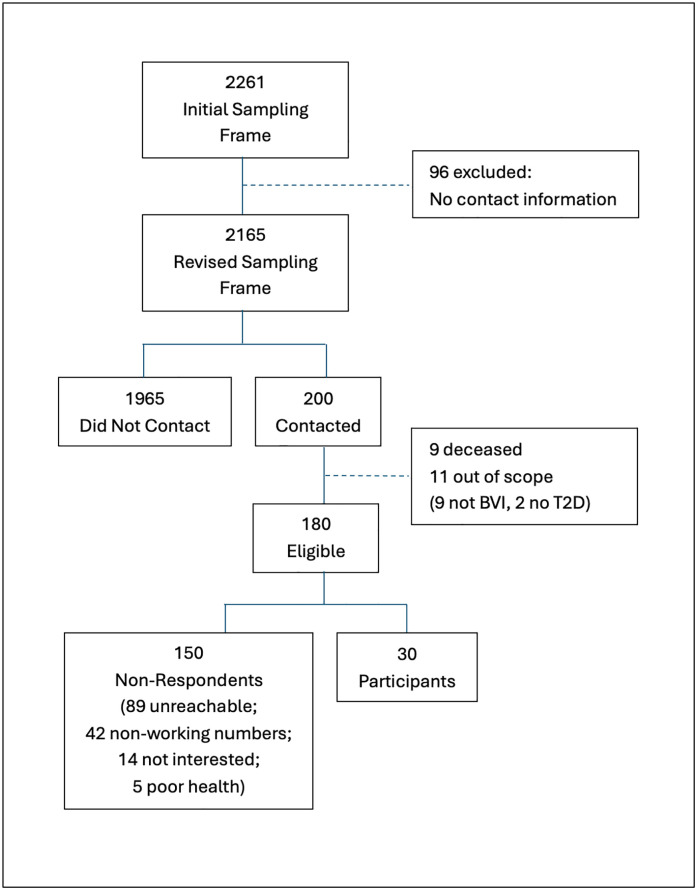
Sample flow diagram (N = 30).

Thirty participants were recruited through Vibrant Works, as current or former clients (Fig 1). Although many individuals met eligibility criteria, enrollment was limited to 30 participants to align with the exploratory objectives of the quantitative phase and to ensure feasibility within the study’s resources and accessibility constraints. Recruitment relied on client records maintained by Vibrant Works, which include diabetes and vision status, ensuring access to the target population. Participants were contacted via phone using information provided by the organization. Inclusion criteria were (a) diagnosis of T2D, (b) blindness or vision impairment documented by Vibrant Works, and (c) ability to provide verbal consent. Exclusion criteria included: (a) no diagnosis of T2D; (b) no diagnosis of blindness/vision impairment, and/or (c) inability to provide verbal consent due to language or health limitations. These criteria were selected to ensure participants could meaningfully engage in the study.

To achieve a sample of 30 participants (a target selected to allow for exploratory bivariate comparisons while remaining feasible for this hard-to-reach population), contact information was provided for 200 randomly selected clients (Fig 1). To minimize selection bias and ensure representativeness of the Vibrant Works client population with T2D and vision loss, we used a simple random sampling approach. This approach provided a transparent and replicable method for participant selection, ensuring equal probability of contact across the sampling frame, reducing systematic bias, and supporting representativeness of this hard-to-reach population. After excluding clients without contact information (n = 96), we generated a revised sampling frame of 2,165 eligible clients. From this frame, we used a computer-generated random number sequence to select clients for recruitment in batches of 25, until we reached our target of 30 participants. In total, 200 clients were contacted, of whom 180 were eligible (20 were ineligible due to being deceased or for not meeting vision or diabetes status criteria), resulting in a response rate of 16.67% (Fig 1).

The sample reflects the composition of Vibrant Works’ client population with T2D, which includes individuals with varying degrees of vision impairment. Of the 30 participants, 10 were blind, and 20 had low vision. All participants provided verbal informed consent using a standardized script outlining the study’s purpose, procedures, risks, benefits, confidentiality, and voluntary participation. Verbal consent was documented in a secure log. The study protocol, including verbal consent procedures, was approved by the University of Texas at San Antonio (IRB FY21-22-255), which granted a waiver of written consent due to the unique barriers experienced by the study population.

### Data collection and procedures

Participants completed a two-part phone-based interview, consisting of (1) a closed-ended portion of the interview on sociodemographic and health-related factors and (2) an audio-recorded open-ended, in-depth interview about their experiences with PA and other aspects of diabetes self-management. Interviews lasted approximately one hour: the closed-ended portion of the interview took participants approximately 30 minutes to complete, and the open-ended portion of the interview lasted approximately 30 minutes on average (mean: 30.92 minutes; range: 8.33–65.38 minutes). Interviews were conducted in English (n = 28) and in Spanish (n = 2), based on participant language preference. Spanish-language interviews were conducted by two bilingual (English-Spanish) researchers trained in qualitative methods. The interview guide and quantitative instruments were translated into Spanish and verified by the two bilingual researchers. All interviews were audio-recorded and transcribed verbatim; Spanish-language interviews were transcribed and translated into English by the bilingual interviewers using a standardized process to ensure consistency across languages.

The *closed-ended questionnaire* included the Exercise Behaviors Scale [31], a six-item instrument assessing minutes of physical activity during the past week. The Exercise Behaviors Scale [31] is part of the measurement set developed for the *Chronic Disease Self-Management Program (CDSMP)* by Lorig and colleagues, which is used to assess adherence to physical activity recommendations and chronic disease self-management programs [32–34]. The Exercise Behaviors Scale assesses the frequency of aerobic exercise and stretching/strengthening activities, with categories converted to estimated weekly minutes of physical activity. The six items of the Exercise Behaviors Scale include: (a) stretching or strengthening exercises, (b) walking for exercise, (c) swimming or aquatic exercise, (d) bicycling (including stationary exercise bikes), (e) other aerobic exercises with equipment, and (f) other aerobic exercises. Participants select none (0), less than 30 minutes per week (1), 30–60 minutes per week (2), 1–3 hours per week (3), or more than 3 hours per week (4) for each category of exercise. Based on these values, the total minutes of PA were computed, and participants were classified as very active (>300 minutes; n = 9), moderately active (150–299 minutes; n = 6), moderately inactive (60–149 minutes; n = 7), and inactive (< 60 minutes; n = 8) [31,35]. Responses were used to estimate the (a) average minutes of weekly PA and (b) associations with meeting PA guidelines (150 +  minutes of weekly PA) [10].

Participant *neighborhood characteristics* were determined based on address using the Area Deprivation Index (ADI), Walk Score^®^, and Transit Score^®^. The ADI is a validated measure of neighborhood disadvantage at the census block level [36,37]. The ADI is based on American Community Survey 5-year data (2019–2023) and is reported as a composite score ranging from 1 (least socioeconomically advantaged) to 100 (most socioeconomically disadvantaged), based on weighted factor scores for 17 indicators [36]. Walkability and public transportation access were assessed using the Walk Score^®^ and Transit Score^®^, publicly available, validated, and widely used metrics for neighborhood walkability and public transportation access [38]. The Walk Score^®^ measures the walkability of an address, access to amenities, and pedestrian friendliness, reported as a composite score ranging from 0 (least walkable) to 100 (most walkable) [38–40]. The Transit Score^®^ measures how well a neighborhood is served by public transit based on frequency, type, and distance, reported as a composite score ranging from 0 (least transit) to 100 (most transit) [38–39].

For the *open-ended qualitative interview*, the interview protocol included several prompts relating to PA and diabetes self-management. Participants were asked: “Could you please speak a bit more about your experiences with PA: What helps? What gets in the way?” and “Is there anything else you would like to add about what would help you manage your diabetes?” All interviews were audio recorded and transcribed verbatim.

### Data analysis

**Fig 2:**
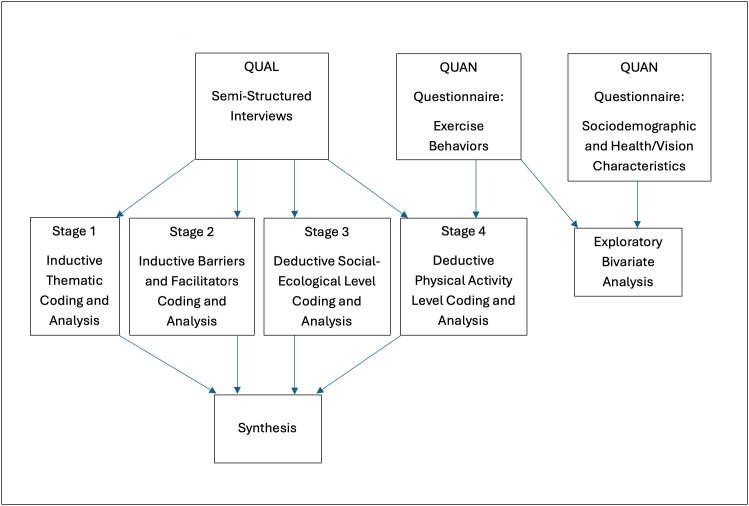
Procedural diagram of study stages: data collection, analytical convergence, synthesis.

A mixed methods approach was used to analyze data. Data were integrated using a convergent mixed methods design, in which quantitative and qualitative data were collected concurrently, analyzed separately, and then integrated during interpretation (Fig 2). Qualitative data were analyzed using an inductive/deductive hybrid thematic analysis [41]. We engaged in a multi-stage coding process, beginning with inductive coding of physical activity experiences and barriers/facilitators (Stages 1–2), followed by deductive coding based on social-ecological level (Stage 3) and physical activity levels derived from qualitative data (Stage 4). Data were integrated through complementarity, using qualitative insights to contextualize and explain quantitative patterns.

A coding scheme was developed inductively from the qualitative data, in consultation with Vibrant Works, to capture (1) PA experiences; and (2) barriers and facilitators to PA (S1 Table). Two researchers independently conducted line-by-line coding of all transcripts. During double-coding, coders met to discuss themes, interpret shared meanings, iteratively review textual patterns, and reach consensus. This collaborative process enhanced rigor and minimized individual bias in qualitative analysis (S1 Table).

Trustworthiness was addressed using established qualitative criteria from Lincoln and Guba [42]. Credibility was enhanced through co-development of the interview guide with the community partner, use of semi-structured interviews, investigator triangulation, piloting with members of the target population, and collaborative interpretation with the community partner. Dependability was supported by a systematic, multi-stage coding process, detailed analytic memoing, and maintenance of an audit trail documenting analytic decisions. Confirmability was strengthened through reflexive team discussions, double-coding, procedures, and grounding all interpretations in verbatim participant quotations. Transferability was supported by providing rich description of the study context, participant characteristics, and methodological procedures, enabling readers to assess applicability to other settings.

Qualitative data were analyzed in NVivo 14 (QSR International Pty Ltd., Release 14.23.2, 2023). Data integration across qualitative and quantitative strands was also conducted in NVivo to facilitate comparison and synthesis of themes with activity-level classification. Quantitative data were entered into StataSE (version 17.0) and analyzed using descriptive and bivariate methods to examine two outcomes: (1) weekly physical activity minutes (continuous) and (2) meeting the weekly PA guideline (binary; 1 if >150 minutes/week). To examine the continuous measure of weekly PA minutes, differences across categorical characteristics were tested using one-way ANOVA. We report omnibus p-values and η² effect sizes. Post hoc tests were exploratory when cell sizes were small. Associations with continuous neighborhood measures were estimated using OLS regression. To examine the binary outcome of meeting PA guidelines (>150 minutes/week), Fisher’s exact tests were used to assess associations with categorical predictors due to small cell counts; two-sided p-values are reported. For continuous predictors (e.g., ADI), ORs reflect the change in odds per unit increase. Effect sizes for this outcome are expressed as Cramér’s *V* (*V*) for categorical variables with more than two levels to provide an omnibus measure of association, while odds ratios (ORs) are reported for binary and continuous predictors. Analyses were prespecified, two-sided, and used an α = .05. Results are interpreted cautiously due to small cell sizes.

## Results

### Sample description

The full list of participants (with pseudonyms, activity levels, and key sociodemographic and health/vision-related characteristics) is shown in Table 1, and overall sample characteristics are shown in Table 2. Participants (ages 44–83; mean: 62.3) were equally male/female. Two-thirds had low vision, and one-third were blind. Seventy percent experienced vision loss due to diabetic retinopathy, with the remainder experiencing vision loss due to another or unknown cause. Reflective of Bexar County’s “minority majority” population status, the sample was also racially and ethnically diverse; 67% of participants self-described their race and ethnicity as Hispanic, Latino, or Mexican American, 20% as African American or Black, 10% as White or non-Hispanic White, and 3% as Asian American. Participants consisted of a geographically diverse sample across greater San Antonio, Texas. Most participants lived in socioeconomically disadvantaged neighborhoods (ADI mean: 71.9, range: 29–99), car-dependent neighborhoods (Walk Score^®^ mean: 38.17; range: 3–81), and transit-limited neighborhoods (Transit Score^®^ mean: 31.27; range: 0–77). PA levels ranged from inactive to very active, with a mean of 181.5 minutes of PA per week. Half of the participants met or exceeded recommended activity levels (Table 2).

**Table 1:** List of study participants.

Name (Pseudonym)	Activity Level	Age Group	Sex	Race and Ethnicity	Vision Status	DR status
Andrés	Very Active	65-74	M	Hispanic	VI	DR
Camille	Very Active	75+	F	NHW	VI	No DR
Celeste	Very Active	75+	F	Hispanic	VI	DR
Clara	Moderately Active	65-74	F	Hispanic	Blind	Unk
Damon	Very Active	65-74	M	Hispanic	VI	No DR
Desmond	Moderately Active	55-64	M	NHB	Blind	DR
Elias	Moderately Active	55-64	M	Hispanic	VI	DR
Enzo	Inactive	55-64	M	Hispanic	VI	No DR
Ewan	Inactive	55-64	M	NHW	VI	DR
Gideon	Very Active	65-74	M	NHW	VI	DR
Hazel	Moderately Inactive	40-54	F	NHB	VI	DR
Hector	Inactive	55-64	M	Hispanic	VI	DR
Hilde	Moderately Active	75+	F	Hispanic	Blind	Unk
Ines	Moderately Active	55-64	F	Hispanic	VI	No DR
Lucia	Moderately Inactive	40-54	F	Hispanic	VI	DR
Miles	Inactive	40-54	M	Hispanic	Blind	DR
Mira	Inactive	75+	F	Hispanic	Blind	No DR
Nico	Moderately Inactive	40-54	M	Hispanic	VI	DR
Nina	Very Active	65-74	F	Hispanic	VI	DR
Noemi	Moderately Inactive	55-64	F	Hispanic	VI	DR
Nora	Inactive	55-64	F	Hispanic	Blind	DR
Owen	Inactive	40-54	M	NHB	VI	No DR
Rafael	Inactive	65-74	M	Hispanic	Blind	DR
Ramona	Very Active	55-64	F	NHB	VI	DR
Salvador	Very Active	65-74	M	Hispanic	VI	No DR
Sabrina	Moderately Inactive	40-54	F	Hispanic	Blind	DR
Soojin	Moderately Active	65-74	F	Asian	VI	DR
Tessa	Moderately Inactive	40-54	F	NHB	VI	DR
Thiago	Very Active	65-74	M	Hispanic	Blind	DR
Victor	Moderately Inactive	55-64	M	NHB	Blind	DR

Abbreviations: Male (M), Female (F), Non-Hispanic Black (NHB), Non-Hispanic White (NHW), Vision impaired (VI), Diabetic retinopathy (DR).

**Table 2:** Sample characteristics (N = 30).

Variable	n(%)
**Physical activity**	
Minutes weekly PA (mean: 181.5; range 0–525)	
Inactive (less than 60 min/wk)	8 (26.67%)
Moderately inactive (60–149 min/wk)	7 (23.33%)
Moderately active (150–299 min/wk)	6 (20%)
Very active (300 + min/wk)	9 (30%)
PA recommendations met	
Yes	15 (50%)
No	15 (50%)
**Sociodemographic**	
Sex	
Male	15 (50%)
Female	15 (50%)
Age (mean: 62.3; range 44–83)	
40-54	7 (23.33%)
55-64	10 (33.33%)
65-74	9 (30%)
75 and above	4 (13.33%)
Race and ethnicity	
Non-Hispanic White	3 (10%)
Hispanic	20 (66.67%)
Non-Hispanic Black	5 (20%)
Non-Hispanic Asian	1 (3.33%)
Household composition	
Live alone	11 (36.67%)
Reside with 1 other person	10 (33.33%)
Reside with 2 + people	9 (30%)
Employment status	
Currently working	1 (3.33%)
Not working due to disability	16 (53.33%)
Retired	12 (40%)
Never worked	1 (3.33%)
**Health and vision status**	
Diagnosed with diabetic retinopathy	
Yes	21 (70%)
No or unknown	9 (30%)
Vision status	
Blind	10 (33.33%)
Vision impaired/low vision	20 (66.67%)
Diabetes duration (mean: 24.8; range 7–45 years)	
25 years or fewer	15 (50%)
More than 25 years	15 (50%)
**Neighborhood factors**	
Area Deprivation Index (mean: 71.9; range 27–99)	
Quintile 1: 1–20	
Quintile 2: 21–40	1 (3.33%)
Quintile 3: 41–60	8 (26.67%)
Quintile 4: 61–80	10 (33.33%)
Quintile 5: 81–100	11 (36.67%)
Neighborhood Walk Score (mean: 38.2; range 3–81)	
90-100: Walker’s paradise	
70-89: Very walkable	3 (10%)
50-69: Somewhat walkable	9 (30%)
25-49: Car dependent	7 (23.33%)
0-24: Very car dependent	11 (36.67%)
Neighborhood Transit Score (mean 31.27; range 0–77)	
90-100: Rider’s paradise	
70-89: Excellent transit	1 (3.33%)
50-69: Good transit	2 (6.67%)
25-49: Some transit	19 (63.33%)
0-24: Minimal transit	8 (26.67%)

### Descriptive and bivariate analyses of physical activity participation

Descriptive and bivariate analyses of group characteristics with (a) weekly PA minutes and (b) percentage meeting PA recommendations of 150 minutes or greater per week are shown in Table 3. Weekly PA minutes differed significantly by age group (*p* = .014, η² = 0.33), with older adults reporting substantially more activity than middle-aged adults. Employment status showed a moderate, nonsignificant effect (*p* = .12, η² = 0.20), with retired participants tending toward higher activity. Neighborhood disadvantage was inversely associated with weekly PA minutes (β = −3.07, *p* = .035), indicating that greater disadvantage was associated with less activity.

**Table 3:** Bivariate analyses of physical activity participation and sample characteristics (N = 30).

	Weekly PA(minutes)			% meeting PA guidelines(>150 minutes/week)		
**Variable**	mean/β (95% CI)	p-value	Effect size (η²/R²)	%/OR (95% CI)	p-value	Effect size (V/OR)
**Sociodemographic**						
Sex		.54	0.01		1.00	0.77
Male	165 (74.98, 255.02)			46.67 (23.29, 71.61)		
Female	198 (128.63, 267.37)			53.33 (28.39, 76.71)		
Age group		.014*	0.33		.002*	0.68
40-54	90 (49.16, 130.84)			0 (Not estimable)		
55-64	128 (39.70, 215.30)			40 (15.11, 71.40)		
65-74	283 (174.03, 392.63)			88.9 (47.76, 98.59)		
75 and above	248 (−28.45, 523.45)			75 (22.05, 96.95)		
Race and ethnicity		.94	0.01		.73	0.26
Non-Hispanic White	220 (−259.12, 699.12)			66.7 (14.04, 96.08)		
Hispanic	184 (111.44, 256.06)			50 (28.60, 71.40)		
Non-Hispanic Black	155 (39.04, 270.96)			33.3 (7.84, 74.61)		
Non-Hispanic Asian	180 (Not estimable)			100 (Not estimable)		
Household Composition		.54	0.04		.18	0.37
Live alone	207 (106.92, 307.62)			63.64 (32.69, 86.31)		
Reside with 1 other person	194 (107.29, 279.71)			60 (28.60, 84.89)		
Reside with 2 + people	137 (10.04, 263.29)			22.2 (5.25, 59.56)		
Employment status		.12	0.20		.013*	0.55
Currently working	240 (Not estimable)			100 (Not estimable)		
Not working (due to disability)	123 (61.72, 183.91)			25 (9.28, 52.05)		
Retired	250 (148.21, 351.79)			75 (43.42, 92.14)		
Never worked	240 (Not estimable)			100 (Not estimable)		
**Health/vision status**						
Diabetic retinopathy		.84	0.002		.43	0.38
Yes	178 (109.26, 246.45)			42.9 (23.33, 64.89)		
No or unknown	190 (87.35, 292.64)			66.7 (32.02, 89.47)		
Vision status		.73	0.07		.70	1.83
Blind	131 (46.46, 214.54)			40 (15.11, 71.40)		
Vision impaired/low vision	207 (136.22, 277.78)			55 (32.77, 75.40)		
Diabetes duration		.38	0.03		.47	2.25
< 25 years	158 (93.28, 222.72)			40 (18.49, 66.21)		
> 25 years	205 (112.57, 297.43)			60 (33.79, 81.51)		
**Neighborhood factors**						
Area Deprivation Index	−3.07 (−5.89, −2.40)	.035*	0.15	−0.03 (−.08, 0.01)	.15	0.97
Walk Score	−0.60 (−2.84, 1.63)	.59	0.01	−0.002 (−0.03, 0.03)	.91	0.998
Transit Score	0.20 (−2.87, 3.28)	.89	0.001	0.01 (−0.03, 0.05)	.55	1.01

Note. P-values for weekly PA minutes were derived from ANOVA or linear regression, with effect sizes reported as η² or R², respectively. For meeting PA guidelines, p-values were based on Fisher’s exact tests (categorical predictors) or logistic regression (continuous predictors), with effect sizes expressed as Cramér’s V (V; variables with >2 categories) or as odds ratios (ORs) for binary or continuous variables. PA = physical activity. *p < .05.

For meeting PA guidelines (>150 minutes/week), age group was a strong and significant predictor (*p* = .002, *V* = 0.68), with older adults substantially more likely to meet recommendations than middle-aged adults. Employment status also demonstrated a large and significant association (*p* = .013, *V* = 0.55), with participants not working due to disability markedly less likely to meet PA guidelines. Neighborhood disadvantage was not significantly associated with meeting PA guidelines. Other sociodemographic, health, and neighborhood factors were nonsignificant.

## Qualitative findings

**Fig 3:**
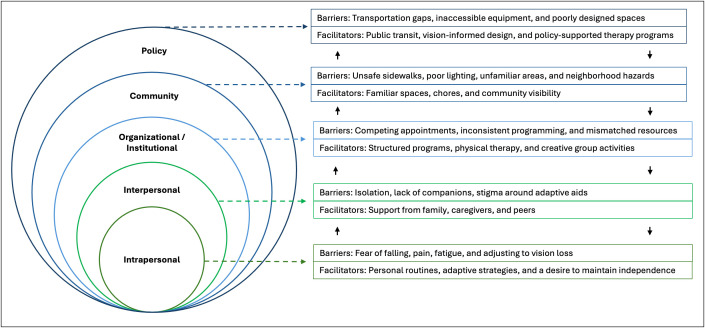
Barriers and facilitators to physical activity according to social-ecological level.

Participants’ accounts of living with T2D and vision loss revealed that PA engagement is a multidimensional experience spanning five social-ecological levels (intrapersonal, interpersonal, organizational/institutional, community, and policy). Specifically, analysis of data resulted in five key themes (with 11 sub-themes) that collectively capture participants’ unique narratives surrounding PA participation, including the barriers and facilitators embedded within these social-ecological domains. The themes that emerged at each of the five social-ecological levels are shown (Fig 3) and discussed below.

### Theme 1. Intrapersonal level – Navigating the inner landscape

Individual factors—such as psychological characteristics and perceptions, or health-related factors—can affect PA opportunities, constraints, and behaviors. At the intrapersonal level, PA was presented by participants as either a mechanism to maintain/regain their independence and mobility, as a risk to their independence and mobility, or—in some cases—as both. While all participants described the more proximal hazards of PA (especially falls), some more active participants emphasized the long-term benefits of PA on their independence, mobility, and health in general. Participants also described difficulties in weighing risks and rewards amid uncertainty (e.g., unknown hazards), vision loss, and other comorbidities or complications. Across these discussions, more active participants emphasized that meaningful exercise progress is built through small yet consistent routines.

#### Concerns, complications, and comorbidities.

All participants, regardless of activity level, identified fear of falling as a key factor limiting their opportunities to engage in PA. Some participants with lower activity levels rarely went out alone and often limited their movement at home due to fear of falling. Participants also described a tension between the perceived benefits and risks of PA; although they recognized that PA could help prevent falls, they were concerned that engaging in PA—particularly in unfamiliar environments—might increase their risk of falling. Numerous comments were made about falls and the desire to limit this risk, including: “there’s very big odds I may fall,” “I hate to say fear, but you know, that’s, that’s a big part of it,” “I don’t want to fall,” and “it [falling] scares me and I think it’s anything, you know, keeps me from being more active it, it’s that.” Desmond (55–64 years, moderately active, blind) described the constant vigilance required when walking outside the home and the mental effort involved in maintaining that level of awareness:

You can get familiar and get a routine. But you never know what’s in front of you when you’re blind. If I step off the curb, there’s very big odds I may fall. But I do stay prepared now because again we have to keep everything in our mind what’s goin’ on, but falls are gonna happen if you’re blind (…). If I wasn’t blind, I could probably briskly walk somewhere (…). I could gracefully walk, but I can’t. I can’t do that. I gotta stop at every corner. I have to stop and listen. Is there a car coming before I cross the street, you know?

A particularly sobering example of fall-related fear was shared by Nora (55–64 years, inactive, blind), who no longer attempts to walk outside after a frightful, near-miss experience:

I ended up on the wrong side, I thought I was gonna be able to cross the street, and I couldn’t cross the street because I have a little bit of sight. (…) I could see the cars coming, but I didn’t know if they were too far back or too close, and I was scared to cross the street. So, I stayed on the other side, and there was no sidewalk. It was just grass, and I couldn’t really see. There was a path, but then afterward, there was no more path, and I panicked because I didn’t know there was gonna be a hole or something I could fall or something. So, yeah, I kinda got scared.

While some participants described themselves as becoming more activity-avoidant over time, other participants have cautiously increased their engagement in PA as they adjusted to vision loss. Participants discussed using adaptive strategies to reduce their likelihood of falling, including activity avoidance, using adaptive aids, slowing movement, proceeding with caution when engaging in activities, and using other senses to perceive danger. Among less active participants, the immediate concern of fall risk is a more prominent factor, while among more active participants, the benefit of PA can drive decisions to participate, despite the risks.

Comorbidities and diabetes-related complications were highly prevalent among participants. In some cases, participants emphasized ways in which PA helped them manage these conditions or as a strategy toward independence. However, the challenges presented by diabetes-related complications and co-occurring conditions should not be understated. Even among more active participants, such as Salvador (65–74 years, very active, low vision), maintaining PA can be challenging alongside the physical discomforts of diabetes-related complications:

The feet hurt; they call it neuropathy. Like the bottom of my feet hurt when I walk, so I have a hard time walking a long way, and then I walk a lot (…) because I’m supposed to. They tell me. Keep walking. Keep walking.

Ramona (55–64 years, very active, low vision) spoke about the additional challenges presented by comorbidities—in this case, arthritis—alongside the challenges of vision loss and diabetes management and noted, “besides the sugar, I have arthritis in my feet, so I’m taking a little more precautions of where I step and where I go to avoid falling.” Some less active participants reported that foot pain due to diabetic neuropathy and diabetic ulcers were key factors preventing them from engaging in PA. In some cases, this restricts PA, and in some cases, it altogether prohibits it. Rafael (65–74 years, inactive, blind) described the desire to walk despite the challenges of pain from diabetic foot ulcers: “I need to try to walk more ‘cause I could at least take 10 steps before my feet start to hurt. I need to do that more every day, but it hurts.” For some participants, foot ulcers required the use of a wheelchair, therefore limiting mobility:

Right now, I have an ulcer on my foot. I’ve had it for three years, and I can’t put any weight on it, so I’ve been in a wheelchair for that time. It makes it difficult. I can’t go walking or anything like that, so.

Frequent medical appointments were experienced as a barrier to PA participation, as participants were managing other chronic health conditions and diabetes-related complications. Mira (75 + years, inactive, blind) shared:

What gets in my way [of exercising] is appointments. It’s not all the time, you know, it’s just some months you’re fine, without appointments, but then sometimes you just have to go every other day, every four days or something like that, and it’s inconvenient, but you never know when something is going to come up. You know, something will happen all of a sudden, and oh shoot, I can’t go and be physical today, I can’t go do this or go do that ‘cause something is going on.

Participants undergoing hemodialysis—a critical treatment for those with diabetes-related end-stage renal disease—described the demanding regimen and treatment-related symptoms, such as fatigue, dehydration, cramping, and headaches, as major barriers to engaging in PA. As most participants were managing multiple health conditions, those demands were perceived as competing priorities that pulled time, resources, and energy away from opportunities to engage in PA.

#### Independence, mobility, and health.

Participants characterized PA as a mechanism through which their independence, mobility, and health could be either maintained or regained over time. Among more active participants, PA was framed as a way in which they could check in with themselves, as an embodied form of independence and mobility. Participants discussed an awareness of the importance for their long-term health and well-being: “I try my best because with my blood pressure and my kidneys, the doctor recommended […] that I have to walk more.” Alongside the disruption of vision loss, participants explained the process and the strategies they employ to achieve progress and goals. Thiago (65–74 years, very active, blind), for example, described his incremental progress in engaging in greater levels of PA to foster confidence in self and ability to be alone for periods of time:

Before, I was like, I won’t move, I won’t move (…) but then I started saying “okay I gotta, I gotta—" and I would ask around me, I said “what what’s here, what’s there, what’s this?” or “How far can I go without, you know, this or that” And, so I have to [inaudible] myself before I felt confidence in wherever I was. That I knew that I didn’t have a, an assistant or somebody guiding me every moment, you know. I could be left alone. I could be left alone for 10, 20 minutes, 30 minutes doing my exercise.

For less active participants, PA was described as a mechanism through which independence can be regained. For example, Lucia reflected on the importance of learning to walk with her prosthetic leg, both to be eligible for a kidney transplant and to regain her prior sense of independence. She discussed a strong motivation to be able to walk around her own living space; she stated:

I used to walk daily (…) when I lost my leg, it stopped a lot of my, well of course, my walking. I just got my prosthetic leg not too long ago (…). Even though I’m blind, even if it takes me feeling the walls, you know, I know the way around my apartment, so I basically want it. That’s what I want in my life, just to walk around my apartment.

#### The pros and cons of mobility aids and adaptive equipment.

PA experiences were nuanced among participants with mobility aids and adaptive equipment. Some participants emphasized that aids/equipment enabled them to be more physically active than they could otherwise, making activities safer. Sabrina (40–54 years, moderately inactive, blind) explained how she uses her white cane (for navigation and detecting obstacles) and her walking cane (for support and balance) to stay mobile and prevent falls:

I’m in constant fear of falling. But when I use my canes to get around, especially when I’m outside the house, they keep me, they’re kind of like my second pair of legs. They help me ’cause I have problems with depth perception too. Like I can see a little bit, but not enough to know what I’m doing sometimes. So, I have to use the canes, and they help me stay mobile, upward I guess. Yeah, so when I first went blind, I was falling a lot.

Clara (65–74 years, moderately active, blind), who also experienced foot pain, described the benefit of using a mobility rollator for outdoor walks so that she could rest if fatigued or if her foot pain became too severe:

You just take [the walker] with you, and you get tired, you just sit there. (….). Before I didn’t [take it on walks] because my feet wasn’t hurting. But it’s been a couple of, it’s been more than a year, I’m like that.

Despite the noted benefits of mobility aids and assistive devices, participants also noted challenges or hesitancy in using them. Participants’ narratives reflected worries about muscle loss, difficulty transporting equipment, and struggling to determine when best to use certain devices over others. Nina (65–74 years, very active, low vision) considered the presence and use of aids as a sort of “slippery slope” to losing her independence. She shared:

I have an issue with certain types of aids, you know, people use aids in the bathroom or (…) to get up out of a chair, or I just feel like the more you rely on those, the less you are going to use your own muscle and your own body to help you do these things, you know? If you lose it, you don’t get it back.

Participants described challenges in selecting which assistive devices to bring, often weighing tradeoffs between safety, convenience, and mobility. Mira (75 + years, inactive, blind) detailed the challenges of not being able to use her rollator, which helps her knee pain, due to difficulty transporting it:

My sister has a vehicle that can’t accommodate a rollator, my daughter either, so (…) I cannot take my rollator; I have to use my cane. And like I say, I’m more secure with my rollator than my cane.

Several participants reported difficulties in choosing among assistive devices, reflecting the competing needs and priorities involved in these decisions. Miles (40–54 years, inactive, blind) described the “different information” provided by different devices, which cannot be simultaneously used:

I used to be able to walk, you know, a distance, and get places, but since I got sick, I’ve had to use my walker. Yes, it’s easier to walk with it, but I can’t go anywhere because I don’t know where I’m going. With the cane, you can get out and say ok, I’m walking, and I’m next to a wall. I know that I can be like a foot away from the wall, because my cane is hitting the wall, but with the walker, you don’t know where you’re at. (…) If you’re walking with a walker, you can tell when the surface changes because the wheels.

This again speaks to the cognitive burden involved with multiple tradeoffs, such as balancing fall risk with PA benefits or device use and selection.

#### Little by little, building physical activity into daily habits and routines.

Participants identified using moderation as a strategy to engage in PA, through building movement into daily habits and routines. For some, this reflects psychological adaptation and acceptance of their changed lives due to vision loss. This also highlights the desire to balance the long-term advantages of PA on their health, well-being, and independence while managing short-term risks that PA can impose. Participants also explained how integrating PA into their home environments and daily routines makes PA more accessible, offering opportunities for independent exercise. Celeste (75 + years, very active, low vision) described the daily structure of small movements throughout her day:

In the morning, I uh get up and (…) move my legs, up and down, sideways, my arms, my legs, when I’m out of bed. Then I get up, go to the restroom, wash myself, get ready for breakfast, and then after I eat, sometimes I’ll walk around. I live on the second floor, so I walk around the building for exercise.

Desmond (55–64 years, moderately active, blind) portrayed PA as a way to “check in” with himself and get the blood flowing:

Being blind, you, still have to be sharp at things ‘cause you don’t ever know what’s coming at you or what’s gonna happen (…) I tend to, not be aggressive when I get up. (…) I just get up first, and I sit up, I stretch, I stand up. Simple. I wouldn’t call it necessarily aerobics, but touch your toes, pullin’ my knees up to me, to my chest, uh, twistin’, hand over head, arms out, uh, and deep breathing is, my routine, pretty much daily. Just to get the blood flowing.

This approach was also emphasized by less active participants. Sabrina (40–54 years, moderately inactive, blind), who uses a wheelchair, characterized her approach to PA as “sit to be fit.” She stated, “I put on YouTube and put dance music on, and I dance kind of in my chair. Trying to get the heart rate up, move my arms.” Even if short bursts of PA are not measurable using our established scales, Enzo (55–64 years, inactive, low vision) shared how small, leisurely movements help with muscle strengthening:

I walk inside the house, I move my legs, so I sit in a chair or on the couch, and I begin to move my legs and arms. I’ve got some weights to lift with my hand. (…) Now I do it more often. (…) I have them on the side of my armchair, and there in the mornings, I listen to the news on TV, and I’m exercising there with the weights. Almost every day.

Participants noted that integrating PA into household chores and home maintenance helped them manage their diabetes while keeping them productive. Nora (55–64 years, inactive, blind) explained that cleaning and other household chores contribute to glycemic control: “Sweeping, and mopping and stuff, and that keeps my sugar levels down.” Gideon (65–74 years, very active, low vision) detailed the home maintenance and chores that help him keep physically active, including mowing, exercising the animals, and home improvement projects:

In the morning, most of the time, I am doing the housework, picking up, or I’m building something in the garage. And during the day, I’m outside doing odds and ends. (…) I do all the mowing (…). In the backyard, we have the animals, we kinda run and play. (…) I’m trying to redo the garage, so I’m picking up stuff and building a few little shelves (…). The other big thing is my mowing because it goes up and down hills (…). Going up is not bad, but going down is a little slowish. I’m happy with what I’m doing so for me, I think this is a good thing.

Of note, Gideon detailed how his home environment enables him to integrate PA throughout the day. As discussed later in this paper, the extent to which home and neighborhood/community environments impose barriers or facilitate PA varied greatly among participants.

### Theme 2. Interpersonal level – Push and pull: Other people as catalysts and constraints

Interpersonal-level factors influencing PA included social support, family dynamics, caregiving, and interactions with others. At the interpersonal level, participants described how other people can help or hinder PA participation. While caregivers, friends, and family members can provide tangible or social support that facilitates PA, other people can also impede PA opportunities. People or their objects can impose risks for safely moving around spaces, and potential interactions regarding vision status or assistive devices can make participants self-conscious or uncomfortable.

#### Unhelpful dynamics and undesired interactions pose roadblocks.

Participants also characterized other people—specifically, sighted people—as barriers to moving freely and engaging in PA. This can relate to clutter in one’s home or to undesired or uncomfortable interactions in public spaces. Desmond (55–64 years, moderately active, blind) discussed mobility challenges within his home environment following marriage, noting that differing preferences for household clutter have limited his ability to navigate safely:

No throw rugs, no area rugs. Not a lot of stuff sitting around (…). Nothing propped up in the doorway (…). I used to be anal about this. I got married, and that’s changed. (…) My wife likes more, more stuff than I do. (…) I used to try to keep mine like that. Where you can walk through without any, anything in your way. (…) So I can maneuver through and not have to worry about tripping over that.

Beyond the home environment, participants described other people as potential obstacles, either physically or through undesired interactions. Thiago (65–74 years, very active, blind) articulated how, on the one hand, a white cane enables him to walk freely and safely, but on the other hand, uncomfortable interactions with others can discourage use of such aids:

I carry my white cane all the time with me, but not everybody knows what it’s for. (…) People think - they feel threatened that you have a cane. You know, some people will feel threatened, some people will understand. “What, what is your problem? Why do you have a cane?” (…). But some people do understand. They move out of the way, and they say, “You need any help, sir?” You know, but not everybody. Not everybody.

Desmond (55–64 years, moderately active, blind), shared that he stays at home largely to avoid interactions with sighted strangers, even if well-meaning: “And then people try to come over and help you, and (…), and people start feeling sorry for you, so just to eliminate the, the [inaudible] in my life, I just stay at home.” While participants differed in how they felt about offers of assistance from strangers, the sentiment was that unexpected or undesired interactions contributed to the avoidance of activity outside the home.

#### Spotters, navigators, and guides provide instrumental support to facilitate physical activity.

Participants detailed the critical role of caregivers—both family members and paid caregivers—in providing instrumental and tangible support for PA participation. Caregivers supported participants by providing transportation and acting as spotters, navigators, or guides, helping them identify obstacles and hazards so they could safely engage in PA. For participants, relying on a caregiver was not characterized as a limitation of their independence, but rather, as a strategy that enabled them to maintain their independence through safe PA and movement. Thiago (65–74 years, very active, blind) elaborated:

I have to have somebody with me ‘cause I am very independent if I know my location and I know my area. (…) Either things change or somebody’s, you know, could be in the way and not be making a noise, and you end up touching them or bumping into them or something. So, I need to always have somebody with me.

For some participants, such Victor (55–64 years, moderately inactive, blind), paid caregivers played a key role in helping them incorporate PA into their daily routines:

I get up every morning and (…) the lady that helps me out is my helper. We get together and we go out to walk. We walk twenty minutes a day. (…) It helped me ‘cause I have a hard time walking. Have to walk with a walker. And so, she kinda helps me (…) with the walker. I’m blind, too, so I can’t really see anything; she helps me out a lot.

Beyond more structured forms of PA, caregivers were also described as essential for helping participants “get the steps in” through other activities, such as shopping. Soojin (65–74 years, moderately active, low vision) described the importance of having a guide for safe movement when running errands:

Like my daughter’s taken me to stores someplace, and if she doesn’t tell me that the ground is unlevel I will fall because I don’t have any depth perception, if that makes any sense so when it’s a little bit dark I can’t tell (…) the difference and I will fall so my daughter, she guides me.

#### Social support through shared activity, partnership, and reciprocity.

Beyond instrumental/tangible forms of support, participants also emphasized the important role of social support in encouraging PA. Participants described social relationships that included activity partners, casual acquaintances, and friendships, which can involve the provision, receipt, or reciprocal forms of social support. Participants shared how having activity partners increases motivation, provides structure, and reduces barriers to PA. While their relationships to these activity partners varied (e.g., spouse or partner, other family member, friend, or neighbor), the benefits were emphasized across participants, regardless of activity levels.

Participants emphasized the reciprocal benefit of PA partnership. Specifically, participants felt motivated by their support systems, enjoyed joint exercise time, and received encouraging “nudges” to engage in activity. Andrés (65–74 years, very active, low vision) described the important and supportive role his wife plays in encouraging his PA, by the simple act of setting weights near his chair and doing the exercises alongside him:

She had a bunch of weights, and she’ll leave them next to me. ‘Cause we have a double recliner, and she’ll sit next to me, and while we’re watching TV and she’ll be doing the exercises too. Little weights and stuff, and I’ll grab ‘em and start moving my arms.

Similarly, Ramona (55–64 years, very active, low vision) noted, “In the afternoons when [my son] comes home, we go out for a while (…) for me, the motivation is that he can move and that I can also move.” Just as participants receive care and support, they also provide it. Tessa (40–54 years, moderately inactive, low vision) described how the provision and receipt of support across generations can help her be more active:

I have a grandson here now, so I take him around the apartment complex and walk him (…) he walks so slow. Like when I pick up the other grandson from school, when I have things to do, my daughter will take me to the grocery store and I try to take my time there, so I can get my walking in (…) She’s an exercise person sometimes I will go to the track with her, it’s kinda rare but I will go with her and get my little two laps around the walking field.

Some participants expressed the benefit of exercising with activity partners with shared lived experience. For example, Clara (65–74 years, moderately active, blind) shared how her group of friends who also have diabetes motivate one another to exercise: “My friends will come over here too, and they, they all have diabetes too, but we all go walking around the building.” Participants also highlighted the benefit of having people who live close by, either to visit during walks or to exercise together. Desmond (55–64 years, moderately active, blind) discussed how he lives close to his mother and noted, “I’ll walk to her, and sometimes she walks back with me. So we both get a little old person exercise.” Activity partners can also play a critical role in identifying or avoiding potential risks or hazards during PA. Elias (55–64 years, moderately active, low vision) conveyed his experiences dancing with his sighted wife: “Sometimes, I don’t see people on my side, and she’ll pull back like a horse, pull the reins back [mimics horse noises] and I’ll stop. That’s how we taught ourselves.”

An important caveat in participants’ narratives was that social support facilitated PA only when such support was available. Unmet needs identified by participants included limited caregiver availability, mismatched schedules, and difficulty building routines. Additionally, some participants’ paid caregivers or transportation providers cannot provide physical assistance with exercise equipment due to liability issues. Finally, participants noted that not having activity partners negatively affected their motivation to engage in PA.

### Theme 3. Institutional/organizational level – Systems that shape activity

Participants identified several ways in which organizations and institutions facilitate or impose barriers to PA. Participants described healthcare and social service providers as instrumental actors in promoting PA when the services and recommendations are tailored and perceived as feasible and useful. Additionally, participants considered community centers and senior centers to be important sites for safe, accessible, and engaging PA.

#### Healthcare and social service providers: Make it useful, doable, and accessible.

Participants described the important roles healthcare and social service providers play in promoting PA: through providing information, resources/equipment, and motivation. Participants also emphasized that these providers best facilitate PA participation when they are met “where they are”, whether that means going to the client’s home or tailoring recommendations to physical, environmental, or practical constraints. Participants appreciated it when providers built on their existing knowledge and experiences to develop PA strategies that suited their needs. Andrés (65–74 years, very active, low vision) explained how his doctor drew upon his past habit of walking (before vision loss) to encourage him to engage in realistic and feasible activities:

They were always asking about what kind of exercises I did (…). And then my vision went out, the less I walked. (…) The doctor said to me, “You’re not walking, you know, don’t just be sitting there. Try some leg lifts! (…) Or get behind the chair and just swing your legs back and forth and to the sides.”

In addition to physicians, participants noted that physical therapy sessions were especially helpful in establishing safe and consistent PA routines. Soojin (65–74 years, moderately active, low vision) explained that these sessions offered a structured environment for PA and helped her develop skills for safely navigating her home: “When I’m doing the exercises, trying to strengthen the muscles, and so I feel like okay, that is helping.”

Participants also shared the need to balance the tradeoffs of seeing doctors and other providers with the inherent challenges of transportation and scheduling demands. Clara (65–74 years, moderately active, blind) shared that her feet had been hurting, which prevented her from walking or engaging in other forms of exercise. She described that these factors make her hesitant to discuss the discomfort with her doctor:

Every time you tell her something, “Well I’m gonna give you a refill so you can go.” And it’s way too far. And sometimes, like my daughter, she might have time to take me, or my sister or somebody, and I don’t want to go.

Mira (75 + years, inactive, blind) echoed the challenges of managing multiple appointments for multiple providers:

It just felt like it was all just happening at the same time. Like I didn’t have any appointments in June or July, and then all of sudden, in August I had to go to my PCP doctor, I went to go see my knee doctor, I went to see my eye doctor and now I have to go get x-rays done for my knee and (…) get dentistry done and then again to the knee doctor. So, it’s like, it’s all happening in one month, a month or two. So, it’s been hard to schedule rides and all that.

Although participants reported benefits from engaging with healthcare providers, including physicians and physical therapists, they faced transportation and scheduling challenges that limited their ability to attend appointments and, in turn, reduced their capacity to participate in PA. While some participants shared a desire to have home-based or virtual visits, this was not available to most participants.

Some participants described the supports and services they received from social service and nonprofit organizations as critical resources for adapting to vision loss and developing strategies for safe PA. Participants found multi-pronged services tailored to blind and low-vision people to be particularly helpful, including in-home and in-community training for using white canes and other equipment such as reflective tape and sunglasses to avoid accidents, injury, and increase comfort. Andrés (65–74 years, very active, low vision) recounted how the tailored supports and training for adapting to vision loss (including in-home white cane training and use of reflective tape) helped him better navigate in his home and community:

[Vibrant Works] had all kinds of training, all kinds of counseling. If I ever needed any help, if I was stressing out because of me losing my vision, they were there for me. (…) [The trainer] actually came by the house and (…) gave me some pointers on how to be able to walk through the house, being able to highlight things and put little reflectors and stuff. At the same time, he taught me to use the cane in the house. Then we went outside and actually walked around the neighborhood.

Andrés further elaborated how training provided by Vibrant Works’ Low Vision Clinic (lighting adjustment and buffering, eyewear) has made it safer for him to exercise outside the home:

They went through about an hour’s worth of details insofar as what kind of lights would help me, what kinds of shades of light were good for me, where I could see something and not see too much of a glare. They actually provided some sunglasses. Ones for indoors, and ones for outdoors. (…) And I also use it insofar as exercise; I’ll go outside in the shadows of the afternoon, and I can see a little better. But enough to go up and down the steps and do a little exercise, stepping up and stepping down. And after a few reps of that, and of course, the more I walk around outside, the more I’m familiar with my yard.

Participants also emphasized the importance of peer support networks that are activated through organizations specializing in blind and low vision populations. Nora (55–64 years, inactive, blind) recounted the monthly peer group meetings that she attends through Vibrant Works. While these meetings don’t necessarily motivate her to engage in more PA, interacting with peers who have also experienced vision loss can provide social support and strategies for those who are interested in more activity:

I go to peer group meetings and, you know, we sit there and, you know, everybody talks about, you know, their problems and stuff like that and what they experience (…). I don’t do as much as they do, you know.

In sum, social service and nonprofit organizations address some key needs that would otherwise be unmet. Organizations focusing on blind and low-vision populations provide tailored, often in-home supports, services, trainings, and equipment to promote safe and accessible PA. Such organizations can also facilitate peer networks and support, an invaluable resource for those who have experienced vision loss.

#### Building community through physical activity: Community and senior centers.

Participants also expressed the importance of community centers and senior centers in fostering safe PA alongside other services and activities. While such activities are not necessarily focused on blind and low vision populations, participants shared that the sense of community and camaraderie motivates them to exercise more. Ramona (55–64 years, very active, low vision) described the resources and motivation she receives by going to the senior center:

They’ll have some line dancing, they’ll have the bootcamp, they have all different types of trainings to try and exercise, but me, I’ll just go get on the rowing machine sometimes and the elliptical. Not the standing one, it’s one for sitting. (…) It’s a group of people, but the equipment is by yourself. (…) There’s people who are about 100 years old and they are doing way better than me. (…) Like, why am I not doing that. And then sometimes I say, that’s not my age group, but it did, it did help a lot.

Celeste (75 + years, very active, low vision) particularly enjoys the bingo-cising classes she participates in at her local senior center. She emphasized the importance of camaraderie and the integration of different physical activities in an enjoyable format:

Bingo-cising is awesome! I would recommend it for anybody, young, old, men, women (…). It’s bingo cards. And they’ll call out like a bingo, sometimes A1, B2, and then if you got it, you cross it and put it down, and then you get up and you walk around for like 15 minutes and then they’ll play another number, another few numbers, and then (…) some exercises, up and down, sideways, put your hands over your head or behind you, turn to the right, turn to the left. And then (…) we’ll walk, and we’ll turn around, clap our hands and then (…) we do that for an hour, an hour and a half. And it makes your body feel so good, makes you feel good, and you’re learning to do other things.

Participants described the group class format as a useful structure for PA because it facilitates support and socialization alongside safe and supervised forms of exercise. Hilde (75 + years, moderately active, blind) also emphasized the accessibility of exercise provided by her local community center:

They call us one at a time to go into the exercise room and we do. There’s a lady that comes out on TV and she comes out stretching and showing you what exercises to do while you are sitting down. And then, sometimes we do the volleyball, and stuff like that (…). We sit on the chair and it’s like we are a team, everybody takes care of everybody else. We’re watching, making sure that we don’t tip over, that we don’t fall and they’re very patient, they’re very good with everybody. I try to do as much as I can. (…) There’s always somebody there watching you (…) We have a lot of support. If we didn’t have them, I don’t know what we would do.

Participants who frequent senior centers and community centers viewed these settings as ideal environments for engaging in safe, enjoyable, and accessible PA. However, not all participants had reliable access to senior centers and community centers. Mira (75 + years, inactive, blind), who lived in a senior living community, stated that fellow residents opted out of classes in their housing community to attend activities at their local senior center, to which she did not have reliable transportation access. As a result, the classes at her senior living community were cancelled due to a lack of interest:

We don’t have exercise classes here because (…) these residents won’t participate (…). So, then they take it away from us. Let’s say we get a person that is willing to come and teach how to do exercises. At first, we have like 9 people sign up, and slowly but surely, we only had 2. So, it was like, you know, the lady that would come to do it would say, I can’t support that, you know?

In summary, senior centers and community centers were described by more active participants as an excellent setting for engaging in PA. Group classes were particularly praised by participants when considered fun, accessible, and facilitating social connections. Less active participants confronted barriers to accessing these community resources and struggled to identify comparable opportunities in other settings.

### Theme 4. Community level – The environment as enabler or obstacle

Across PA levels, participants detailed challenges accessing community resources for PA, including fall risks, lack of social support, and transportation barriers. Some participants were successful in utilizing community-level supports and resources for PA purposes, such as senior and community centers described above. Participants also articulated challenges aligning resources to engage in PA, a problem amplified by accessibility barriers.

#### Challenges in aligning resources to engage in physical activity.

Participants depicted community resources, such as parks/green spaces, as invaluable settings for engaging in PA. However, for most participants, these resources could not be accessed directly and required the coordination of assistance from caregivers or transportation providers to utilize. For example, Ramona (55–64 years, very active, low vision) identified a park near her home where she walks with family. Despite its proximity to her home, she must rely on the availability of family to transport and accompany her:

We have a park nearby, and that’s where we go to walk (…). It’s a park, and it has like a quarter mile to walk around. (…) We can’t [walk there]; it’s very much on the road. We go to the park, park the car, and then walk.

This experience is echoed by Nico (40–54 years, moderately inactive, low vision), who would like to go outside the home for exercise and other social activities independently. However, transportation barriers make it challenging for him to get from place to place. Attempts to leave the home can be frustrating and make him feel dependent on others:

There’s a park, but I would have to walk a distance (…), the YMCA or whatever is still far. I would have to walk a couple of streets down. (…) The walking is what does it for me. And last time I was just walking to a friend’s party, birthday party, in order to make it on time, I left at 12:00, like 12:30, and after that time I got tired and turned back and sat at the bus stop and called my mom to pick me up.

These excerpts speak to a broader issue of person-environment fit that emerged across social-ecological levels: participants could theoretically engage in PA, but environmental obstacles prevented them from doing so. The need for assistance and support (e.g., accessible transportation or guides to avoid hazards) affects opportunities, and in some cases, underscores the lack of autonomy participants feel.

#### Community accessibility barriers to physical activity.

As articulated across preceding social-ecological levels, participants expressed concerns about hazards in their communities, preventing them from engaging in PA. Such hazards include the absence of sidewalks or poor sidewalk quality, the absence of curb cuts, poor lighting, the presence of vehicles or other pedestrians, bad weather, crime, or loose dogs. This was a limiting factor to community PA participation across all participants, from very active to inactive. Even the participants who live in highly walkable parts of the city with good sidewalk access, such as Damon (65–74 years, very active, low vision), described sidewalk quality as a factor limiting his opportunities to exercise outside the home: “I try to stay away from the sidewalks because of a lot of the cracks. I have a tendency of falling down.”

Inconsistencies in marking and the lack of visual cues in the built environment were among the accessibility barriers discussed by participants, such as Elias (55–64 years, moderately active, low vision):

When they have steps that they got, that square tiles and they’re all brown, they don’t look like stairs, they look like ramps, and especially if there’s nothing to hold onto, that’s when it gets tough. You start understanding how people with impairments—you know, handicapped—like I got a handicap permit for parking. It’s not that- my legs are fine. If I had to run, I could run, but if there’s a hole I’m going down, ya know.

The accessibility barriers experienced by participants, as well as challenges in aligning resources to overcome these barriers, constitute significant challenges for participants to engage in PA. The challenges to the person-environment fit are poignantly characterized by Miles (40–54 years, inactive, blind), who conveyed his desire to go outside the home and exercise, despite the barriers he encounters:

Some have the little walkways, some don’t. (…). There should be a lot of different things that could help people. To make it easier for people that are blind, ‘cause not all of us want to stay home. If it would be safer for us to go somewhere, I’m pretty sure a lot of us would leave the house to go do stuff, exercise, you know.

While participants may feel safer engaging in PA within the home environment, the desire (often unmet) to exercise outside the home was characteristic of misalignment in person-environment fit. Opportunities for being physically active outside the home were missed due to accessibility problems and other barriers in the neighborhood and community environments.

### Theme 5. Policy level – Infrastructure and access at scale: Patching together resources

Policy-level factors, including laws and regulations, shape health behaviors and outcomes. Across all participants, policy-level factors emerged as key determinants of PA opportunities, operating at intrapersonal, interpersonal, organizational, and community levels. For example, participants detailed how factors related to the built environment (sidewalk quality and availability, curb lines and other accessibility markers) and community resources (access to transportation, community and senior center programs, social services) affected their opportunities to engage in PA and safely navigate in their neighborhoods and communities. However, few participants spoke directly about the policy landscape affecting their opportunities to engage in PA.

Elias (55–64 years, moderately active, low vision), discussed the role of local political decision-making underlying accessibility concerns: “They’re spending money on different, other stuff, and it’s our councilmen too, the district, you know that’s what they gotta do (…) people don’t understand it until you’re there.” He then shared how, in the absence of broader public supports, he has mobilized his personal and interpersonal resources to make PA happen: getting rides, activating support networks, and avoiding situations and circumstances where he could be at risk of a fall or other accident or injury. As described above, participants who were not active emphasized challenges in patching together these resources. More active participants developed ways to work PA into their daily lives—within the safe and controllable context of their home environments—while others utilized community supports and social services to broaden their opportunities for PA engagement.

## Discussion

This study is the first to examine barriers and facilitators to PA among adults living with both T2D and vision loss, addressing a critical gap in understanding determinants of activity in this population. Our findings highlight the complex interplay of intrapersonal, interpersonal, organizational, community, and policy-level factors that shape opportunities for PA.

Using a convergent design and an inductive-deductive hybrid thematic analysis, we employed complementarity to enrich interpretation. Qualitative data added context and depth to quantitative classifications, revealing how individual experiences and environmental factors shaped PA levels. This approach captured both measurable activity patterns and the lived experiences underlying them, underscoring the value of mixed methods for examining complex health behaviors in populations with disabilities.

Aligned with the Social-Ecological Model [25], participants identified factors influencing PA at each ecological level. We identified 5 overarching themes and 11 subthemes (Table 4) characterized through the domains of the Social Ecological Model: intrapersonal (e.g., fear of falling, comorbidities), interpersonal (e.g., social support, caregiving), organizational (e.g., healthcare and community programs), community (e.g., neighborhood accessibility, transportation), and policy (e.g., infrastructure and resource allocation).

**Table 4:** Social-ecological level and qualitative themes/subthemes.

**Intrapersonal level** **Theme 1. Navigating the inner landscape** Concerns, complications, and comorbidities Independence, mobility, and health The pros and cons of mobility aids and adaptive equipment Little by little, building physical activity into daily habits and routines **Interpersonal level** **Theme 2. Push and pull: Other people as catalysts and constraints** Unhelpful dynamics and undesired interactions pose roadblocks Spotters, navigators, and guides provide instrumental support to facilitate physical activity Social support through shared activity, partnership, and reciprocity **Institutional/organizational level** **Theme 3. Systems that shape activity** Healthcare and social service providers: make it useful, doable, and accessible Building community through physical activity: community and senior centers **Community level** **Theme 4. The Environment as enabler or obstacle** Challenges in aligning resources to engage in physical activity Community accessibility barriers to physical activity **Policy level** **Theme 5. Infrastructure and access at scale: Patching together resources**

*Intrapersonal* barriers to PA included fear of falling, pain, fatigue, and adjustment to vision loss, while facilitators encompassed personal routines, adaptive strategies, and a desire for independence. These factors interacted across social-ecological levels to shape PA opportunities and constraints (Fig 3). Fear of falling, reported by all participants, was a major barrier to PA. These qualitative findings align with prior quantitative evidence linking fear of falling to PA among older adults with sight loss [43] and T2D [44]. Past research shows that fear of falling is significantly associated with limitations in self-care, challenges with mobility [45], and more severe depression and anxiety [46]. Fear of falling may reduce confidence in balance, which in turn decreases willingness to engage in PA [47]. To promote PA and address these intrapersonal barriers, practitioners should offer tailored balance and strength training approaches that promote falls awareness. Further, modular “little-by-little” routines can be adapted around comorbidity-related fatigue and scheduling challenges, thereby offering client-centered approaches and treatment targets.

*Interpersonal* barriers included isolation, lack of companions, and stigma/complications with adaptive aids, while facilitators included support from family, caregivers, and peers. Family and peer support heavily influenced opportunities for, and experiences of, PA [26]. Additionally, participants stressed that greater access to peer support (including support from those who have also experienced vision loss) would encourage them to be more physically active, consistent with findings from past studies [48–51]. PA promotion strategies can leverage existing social and instrumental support structures while also fostering the development of new supports. For example, peer-led activity groups (whether in-person or virtual) can normalize adaptive strategies, build community, and enhance motivation. Specific training should be tailored to caregivers as well, with a focus on spotting and assisting with safe navigation. As negative public perceptions can deter activity, awareness campaigns can improve access to public spaces for blind/low vision individuals [26,45,52].

*Organizational* *and* institutional barriers included competing appointments, inconsistent programming, and mismatched resources, while facilitators included accessible, structured programs, physical therapy, and creative group activities. Additional resources or options that enhance flexibility should be provided for medical appointments, including telehealth services and home-based physical therapy, to reduce scheduling and transportation barriers [28]. Tailored programs and services, including Orientation and Mobility (O&M) training, were identified as critical for adjusting to vision loss and regaining mobility and independence [21,53]. Access to such services should be expanded, ideally offered in the home and community environments. Multi-pronged approaches tailored to those with T2D and vision loss (e.g., the integration of O&M training, tailored PT, and diabetes education) might expand access. Further, access to adaptive equipment and opportunities for safe and supported PA in gyms, fitness centers, senior centers, and community centers should be expanded, ideally, with spotting support/assistance available when needed [50,51,54].

*Community* and *policy* barriers included unsafe sidewalks, poor lighting, neighborhood hazards, transportation gaps, inaccessible equipment, and poorly designed spaces. Broader facilitators included access to familiar and accessible spaces, community visibility, accessible public transit, vision-informed design, and policy-supported therapy programs. To enhance opportunities for safe PA, Americans with Disabilities Act (ADA) standards should be prioritized and proactively upheld in the built environment, and further investments in transportation and car-free/walkable spaces are needed [52,53,55]. Bivariate analyses showed no significant link between walkability/transit access and PA, likely due to the measure not capturing safety and accessibility for blind/low vision populations. Walkability and transportation metrics should be updated to address accessibility for people with disabilities. While disability-specific programs are essential for addressing unique needs, universal design approaches are critical to ensure environments and services are accessible to all. Public spaces should be co-designed with blind/low-vision stakeholders to enhance accessibility through clear wayfinding, tactile cues, maintained sidewalks, and escorted access to parks/centers. Accessible physical activity initiatives, including home-based exercise programs, telehealth-supported physical therapy, and community partnerships, represent scalable approaches that can bridge gaps in care for individuals with vision loss and other chronic conditions. Greater resources are needed to address inequities in access to PA infrastructure and public spaces more broadly.

Our data also extend the Social Ecological Model, pointing to cross-level intersections of disability and chronic disease. For example, the intrapersonal challenge of falls and fear of falling was amplified by social and community-level factors such as inaccessible infrastructure and poor sidewalk conditions, limiting safe spaces for PA. Quantitative findings reinforced this, with participants in lower ADI neighborhoods engaging in significantly fewer PA minutes. While only one participant cited financial limitations directly as a PA barrier, access to equipment and safe home environments were constrained by socioeconomic factors, further supported by the ADI-PA association [36,37]. This may suggest that environmental barriers amplify intrapersonal constraints. Social and tangible support—such as the availability of caregivers for navigation or activity partnership—mitigated these barriers for some participants, in line with prior studies [22,23].

**Fig 4:**
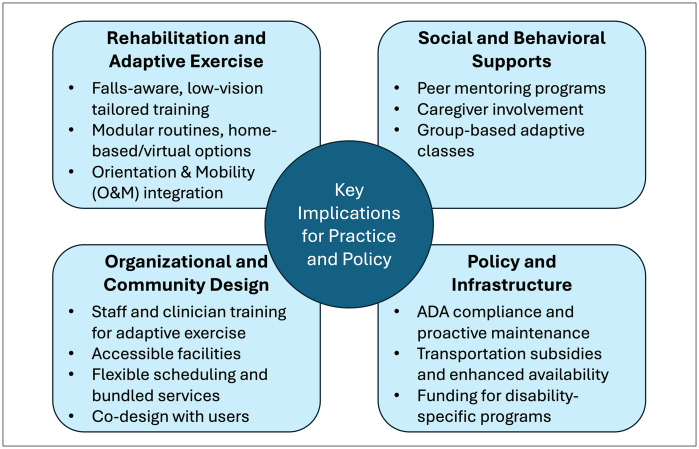
Key implications for practice and policy.

Several implications for practice and policy can be gleaned from these findings that transcend each social-ecological domain (Fig 4). First, accessibility emerges as a cross-level modifier. Accessibility features (or their absence)—including assistive devices and O&M training (organizational) to tactile paving and curb ramps (community/policy)—operate as modifiers of intrapersonal determinants such as fear of falling or confidence. This suggests an “accessibility pathway” that traverses social-ecological levels: when accessibility supports are present, intrapersonal fears are mitigated; when absent, fears are amplified [23,24,53,55]. Second, caregiving/spotting serves as a bridging mechanism. Interpersonal supports function as bridges between intrapersonal motivation and organizational/community opportunities. Caregivers and activity partners reduce environmental uncertainty (hazard detection, navigation) and translate recommendations into feasible routines—highlighting the dependence of intrapersonal change on social scaffolding in disability contexts [22,23,26,49,50]. Third, participants vividly described person-environment mismatches (e.g., “walkable” but not navigable), prompting adaptive strategies (home-based micro-bouts, assistive technologies, timing to avoid glare) that maintain participation despite barriers.

Beyond diabetes care, these strategies align with broader goals for individuals managing multiple chronic conditions or recovering from ICU stays—where mobility limitations and sensory impairments often intersect—to support independence and functional capacity. For example, modular (“little-by-little”) routines and caregiver-supported navigation can be adapted for hospital-to-home transitions, ensuring patient-centered discharge planning. Similarly, accessible community programs—such as senior centers and peer-led activity groups—mirror best practices in rehabilitation by combining physical activity with social engagement and environmental safety. This extends the Social Ecological Model by foregrounding adaptive, disability-informed behaviors as dynamic responses to structural constraints [26,52,56,57].

Several limitations should be considered when assessing the implications of the study. In the quantitative portion, the sample size is small (N = 30), which limited statistical power for multivariate modeling and subgroup analyses. To mitigate the likelihood of unstable estimates in categories with few participants, we utilized exact statistical methods and omnibus effect sizes (Cramér’s *V*) for bivariate comparisons. While these methods are appropriate for an exploratory analysis of this hard-to-reach population, they possess inherent trade-offs. Fisher’s exact test is inherently conservative, which may increase the risk of Type II errors in a small sample. Furthermore, Cramér’s *V* can be sensitive to table dimensions and empty cells, which may impact the stability of effect size estimates in categories with very low counts. Consequently, these findings should be interpreted with caution and viewed as a basis for future research rather than as definite population-level estimates.

The Exercise Behaviors Scale relies on self-reported frequency of exercise, which may introduce recall and social desirability bias, potentially inflating estimates of activity levels. While prior studies have found high internal consistency for related CDSMP scales, such as self-efficacy (Cronbach’s α ≈ .91) [31], no published factor-analytic studies have confirmed dimensionality of the Exercise Behaviors Scale specifically or assessed measurement invariance across populations. Further studies should validate the subscale in larger, more diverse samples, including reliability testing and confirmatory factor analysis, to strengthen its use as a standalone instrument.

Further, there are limitations on the generalizability of these study findings due to the nature of the sample (urban, predominantly Hispanic). San Antonio is also among the most income-segregated cities in the United States [8,9], and ADI relationships found here may not be observed in cities with less pronounced neighborhood-level income gaps. Recruitment from a single community-based organization may limit generalizability to adults with T2D and vision loss who are not connected to similar services or who live in different geographic or cultural contexts. The response rate was 16.7% (30 of 180 eligible participants), which introduces potential non-response bias and limits generalizability. Individuals who agreed to participate may differ systematically from those who declined or were unreachable, for example, in health status, functional ability, or access to resources. As a result, findings may overrepresent individuals who are more engaged with community services, have higher levels of motivation to discuss diabetes self-management, or are generally more able and willing to participate in research. While this sample reflects the demographic composition of the eligible organization client population, caution should be exercised in extrapolating results to broader populations of adults with T2D and vision loss, particularly those not connected to community-based organizations.

The prevalence of T2D and vision loss continues to rise nationally and regionally. In the United States, 14.7% of adults have T2D [1], and 2.2% experience significant vision loss [2]. In Bexar County, diabetes prevalence reaches 15% [5] and vision loss prevalence exceeds 3% [6]. These trends underscore the urgent need for targeted interventions both locally and nationally. Public health strategies should prioritize inclusive design, caregiver support, and community resources to reduce disparities and improve diabetes self-management among individuals with vision impairment. Future research should examine scalable models such as telehealth and home-based exercise programs, as well as policy initiatives that enhance accessibility in the built environment and expand adaptive physical activity programs. Addressing these gaps is critical to mitigating the disproportionate burden of diabetes-related vision loss and promoting health equity in underserved communities.

## Supporting information

S1 Table Qualitative coding scheme and process. (PDF)
