# Supplementary material for: “You never know what’s in front of you”: A mixed methods study of barriers and facilitators to physical activity among blind and low-vision adults with type 2 diabetes
Source: PLoS One. 2026 Jun 17;21(6):e0332565. doi: 10.1371/journal.pone.0332565 (PMC13274851; doi:10.1371/journal.pone.0332565)
Supplement: S1 Table — (PDF) [file pone.0332565.s001.pdf]

S1 Table. Qualitative Coding Scheme and Process

**Stage 1 Codes and Subcodes: Physical Activity Experiences (Inductive)**	
**Access and Accessibility**<br>Falling and Fear of Falling<br>Physical Activity Accessibility/Equipment<br>**Attitudes, Beliefs, and Coping**<br>Acceptance, Confidence, Motivation<br>Habits and Rituals<br>Independence/Lack of Independence<br>Moderation<br>Pain and Injury<br>Preferences, Likes/Dislikes<br>**Diabetes-Related Knowledge/Experience**<br>Pre-diagnosis Physical Activity<br>Post-diagnosis Physical Activity<br>**Financial and Other Resources**<br>Cost/Access to Benefits and Social Services<br>Time Constraints and Availability for PA	**Health and Wellness**<br>Comorbid Conditions<br>Mental and Emotional Health<br>Health Care<br>**Identity, Language, and Culture**<br>Age<br>Identity as Disabled<br>**Social Connection and Support**<br>Family Dynamics/Household Composition<br>Social Isolation, Support, Relationships<br>Pets or Service Animals<br>Tangible Supports and Caregiving<br>**Neighborhood**<br>Transportation Access<br>Walkability/Sidewalks<br>Safety
**Stage 2 Codes: Barriers and Facilitators to Physical Activity (Inductive)**	
Barrier to Physical Activity	Facilitator to Physical Activity
**Stage 3 Codes: Social-Ecological Levels (Deductive; theory-based)**	
Intrapersonal<br>Interpersonal<br>Organizational<br>Community<br>Public Policy	
**Stage 4 Codes: Physical Activity Levels (Deductive-quantitative observation-based)**	
Inactive (less than 60 min/week)<br>Moderately inactive (60-149 min/week)<br>Moderately active (150-299 min/week)<br>Very Active (300+ min/week)	
