# Supplementary material for: “You never know what’s in front of you”: A mixed methods study of barriers and facilitators to physical activity among blind and low-vision adults with type 2 diabetes
Source: PLoS One. 2026 Jun 17;21(6):e0332565. doi: 10.1371/journal.pone.0332565 (PMC13274851; doi:10.1371/journal.pone.0332565)
Supplement: Attachment — Submitted filename: Comments.docx [file pone.0332565.s002.docx]

The study adopts a mixed-methods approach grounded in the Social Ecological Model, contributing valuable insights for inclusive diabetes care and physical activity promotion. However, certain methodological, interpretive, and theoretical aspects require clarification and refinement to strengthen rigor, reproducibility, and conceptual linkage.

Major Issues

The quantitative component (Exercise Behaviors Scale) requires more detail: scoring, validity, and how results informed the qualitative phase.

Sampling strategy is insufficiently justified — the paper should clarify recruitment channels, inclusion/exclusion criteria, and representativeness of blind vs. low-vision participants.

Data integration strategy in the mixed-methods design (e.g., triangulation or complementarity) needs explicit description in Methods or Discussion.

Theoretical Integration

While the Social Ecological Model is cited, its operationalization is inconsistent. Authors should map each theme and subtheme directly to model domains (intrapersonal, interpersonal, community, policy) to strengthen conceptual rigor.

Discussion should elaborate how findings inform or extend this framework in disability and diabetes contexts.

The discussion focuses on descriptive barriers/facilitators but lacks connection to multidisciplinary rehabilitation, adaptive exercise, or policy implications. Integrating previous evidence could emphasize how structured physical activity frameworks can bridge psychosocial and physical challenges in chronic disability contexts. Add below phrase ….

“Previous research has emphasized the rehabilitative and preventive value of structured physical activity programs in promoting both mental and physical well-being among individuals with chronic disabilities [Nasif et al., 2025, <https://doi.org/10.31579/2578-8868/359> ]. Building on this perspective, our study helps extend this framework by identifying context-specific barriers and facilitators that can inform adaptive exercise models and inclusive rehabilitation strategies for adults with visual impairment and type 2 diabetes.”
