# Supplementary material for: “You never know what’s in front of you”: A mixed methods study of barriers and facilitators to physical activity among blind and low-vision adults with type 2 diabetes
Source: PLoS One. 2026 Jun 17;21(6):e0332565. doi: 10.1371/journal.pone.0332565 (PMC13274851; doi:10.1371/journal.pone.0332565)
Supplement: Attachment — Submitted filename: Response to Reviewers 12.16.25.docx [file pone.0332565.s003.docx]

December 16, 2025

Maheshkumar Baladaniya, PT, MS, DPT
Academic Editor, *PLOS ONE*

Response to Reviewers

RE: “**You never know what’s in front of you”: A mixed methods study of barriers and facilitators to physical activity among blind and low vision adults with type 2 diabetes** (Submission ID: PONE-D-25-47274)

Dear Dr. Maheshkumar Baladaniya,

The manuscript has been revised in accordance with the anonymous reviewers’ suggestions. I appreciate the editor’s and reviewers’ thoughtful guidance and expertise on the manuscript. I have endeavored to be forthcoming and cooperative in addressing this constructive feedback and preparing the revised version of the manuscript.

Below, I include the suggestions from each reviewer and include the page number and lines where corresponding revisions are located in the revised, unmarked version of the manuscript. Changes are shown in the marked-up version (‘Revised Manuscript with Track Changes’), per editorial instructions. Revisions have also been made based on editorial instructions.

Thank you for the opportunity to revise this manuscript, which is being considered for publication in *PLOS ONE.*

Sincerely,

Emily J. Nicklett, PhD, MSW (Corresponding Author)

Associate Professor of Social Work and Public Health

College for Health, Community and Policy

University of Texas at San Antonio

Email: [Emily.Nicklett@utsa.edu](mailto:Emily.Nicklett@utsa.edu)

Editorial Comments:

1. *Please ensure that the manuscript meets PLOS ONE’s style requirements, including those for file naming.*

Thank you for this suggestion. We have reviewed the documents and made minor changes to align with the style requirements, including:

- The filename for S1 Table has been changed to S1_Table.
- The Figure names now include “.tiff” as part of the name (e.g., Fig1 changed to Fig1.tiff).
- Line numbers are now included in the title page.
- The corresponding author’s email address is no longer underlined/hyperlinked and now aligns with the style requirements.

2. *The grant information provided in the ‘Funding Information’ and ‘Financial Disclosure’ Sections do not match. Please ensure that you provide the correct grant numbers for the awards you received for your study in the ‘Funding Information’ section.*

The funding information is now consistent, and the discrepancies have been removed.

3. *In the Competing Interests section, please confirm that the stated competing interests does not alter your adherence to all PLOS ONE policies on sharing data and materials, by including the following statement: “This does not alter our adherence to PLOS ONE policies on sharing data and materials. If there are restrictions on the sharing of data and/or materials, please state these. Please include this in your cover letter and it will be changed in the online submission form on your behalf.*

We confirm that the competing interests do not alter adherence to PLOS ONE policies on sharing data and materials. We also identify our restrictions on data sharing. In response to this request, we include this information in our cover letter, with the request that it be changed in the online submission form on our behalf.

4. *Please include your full ethics statement in the ‘Methods’ section of your manuscript file. In your statement, please include the full name of the IRB or ethics committee who approved or waived your study, as well as whether or not you obtained informed written or verbal consent. If consent was waived for your study, please include this information in your statement as well.*

This information is included in the ‘Participant recruitment’ section of the ‘Methods’ section of the manuscript (page 7, lines 148-153).

5. *We note that you have indicated that there are restrictions to data sharing for this study. For studies involving human participant data or other sensitive data, we encourage authors to share de-identified or anonymized data. However, when data cannot be publicly shared for ethical reasons, we allow authors to make their data sets available upon request. […] Before we proceed, please address the following prompts:*
(a) *If there are any ethical or legal restrictions on sharing a de-identified data set, please explain them in detail (e.g., data contain potentially identifying or sensitive patient information, data are owned by a third-party organization, etc.) and who has imposed them (e.g., a Research Ethics Committee or Institutional Review Board, etc.). Please also provide contact information for a data access committee, ethics committee, or other institutional body to which data requests may be sent.*

The Institutional Review Board at the University of Texas at San Antonio has restricted sharing data, even if de-identified, publicly or upon request. Per the UTSA IRB (irb@utsa.edu), data sharing conflicts with the agreement provided to participants in the consent form for the study:

“Data will be kept in a secure, password-protected database and will only be accessed by members of the study team. Data will be kept for 3 years after the study is complete and will be destroyed after that time.”

The rationale for this approach was that it contains potentially identifying information for a geographically specific and potentially vulnerable study population, and that added protections were therefore needed.

Our COI statement now includes the following statement:
“In accordance with the University of Texas at San Antonio Institutional Review Board (irb@utsa.edu), and the terms of participant consent, the qualitative and quantitative datasets underlying this study cannot be shared publicly, as doing so would risk breaching participant confidentiality. Summary data and results are provided within the manuscript.”

(b) *If there are no restrictions, please upload the minimal anonymized data set necessary to replicate your study findings to a stable, public repository and provide us with the relevant URLs, DOIs, or accession numbers.*

*Please update your Data Availability statement in the submission form accordingly.*

The Data Availability statement has been updated accordingly.

6. *Please remove all personal information, ensure that the data shared are in accordance with participant consent, and re-upload a fully anonymized data set.*

We appreciate this suggestion. As the exact ages of participants included in the manuscript constitute identifying information, we have replaced the exact ages in Table 1 and throughout the manuscript with age ranges: 40-54, 55-64, 65-74, and 75+ and removed the ‘Household composition’ column. As we are not permitted to publish the data set (per University of Texas at San Antonio IRB) due to potential violation of participant consent processes, we unfortunately cannot upload or provide an anonymous dataset beyond what is shared in Table 1.

7. *If the reviewer comments include a recommendation to cite specific previously published works, please review and evaluate these publications to determine whether they are relevant and should be cited. There is no requirement to cite these works unless the editor has indicated otherwise.*

We appreciate this suggestion and proceeded accordingly.

8. Please review your reference list to ensure that it is complete and correct.

We have reviewed our reference list, made corrections, and confirm that it is complete and correct.

Reviewer 1:

*Major Issues*

1. *The quantitative component (Exercise Behaviors Scale) requires more detail: scoring, validity, and how results informed the qualitative phase*

We appreciate this helpful suggestion. The ‘Data collection and procedures’ and ‘Limitations’ sections provide additional detail about the scoring and validity of the Exercise Behaviors Scale (page 8, lines 163-179; page 42, lines 969-976).

In these sections, we identify the scale part of the Chronic Disease Self-Management Program (CDSMP). The scale is widely used in CDSMP evaluations and research (e.g., CDC, 2013; Wilmoth et al., 2020). While the exercise behaviors-specific subscale has not been formally validated, internal consistency for other subscales in the CDSMP (e.g., self-efficacy) was high (Cronbach’s α ≈ .91). For the overall CDSMP, psychometric testing was conducted on a sample of 605 adults with chronic conditions, demonstrating acceptable reliability and responsiveness to change. The exercise behavior items specifically showed significant improvements at 4-6 months and 9-12 months post-intervention, supporting construct validity (Lorig et al. 2001). Subsequent meta-analyses and program evaluations have confirmed the sensitivity of these items to behavioral change across diverse populations, including older adults and people with disabilities, although no standalone factor analysis validation of the subscale has been published.

We revised the ‘Data analysis’ section to more clearly discuss how the Exercise Behaviors Scale informed that qualitative phase (pages 10-11, lines 216-229; Fig 2: Stage 4; S1 Table: Stage 4).

2. *The sampling strategy is insufficiently justified—the paper should clarify recruitment channels, inclusion/exclusion criteria, and representativeness of blind vs. low-vision participants.*

Thank you for this valuable feedback. We have revised the manuscript to provide greater clarity and justification for the sampling strategy (pages 6-7, lines 124-153; Fig 1):

(a) Recruitment Channels
    We now explicitly state that participants were recruited through Vibrant Works, a nonprofit organization serving individuals who are blind or vision impaired. Recruitment relied on client records maintained by Vibrant Works, which include vision and diabetes status, ensuring access to the target population. Participants were contacted via phone using information provided by the organization.
(b) Inclusion and Exclusion Criteria
    We have clarified that the inclusion criteria were (a) diagnosis of type 2 diabetes (T2D), (b) blindness or vision impairment documented by Vibrant Works, and (c) ability to provide verbal consent. Exclusion criteria included: (a) no diagnosis of T2D or blindness/vision impairment, or (b) inability to provide verbal consent due to language or health limitations. These criteria were selected to ensure participants could meaningfully engage in the study.
(c) Representative of Blind vs. Low-Vision Participants
    We have added a statement including that the sample reflects the composition of Vibrant Works’ client population, which includes individuals with varying degrees of vision impairment. Specifically, of the 30 participants, 10 were blind and 20 had low vision, consistent with organizational records. This clarification addresses representativeness within the study population.
(d) Justification of Sample Size
    We have included a rationale for the target sample size of 30 participants, noting that the number was selected to enable adequately powered bivariate comparisons while considering feasibility given recruitment challenges in this population.

These revisions appear in the ‘Participant Recruitment’ section of the manuscript (pages 6-7, lines 124-153). We believe these changes address the reviewer’s concerns and strengthen the transparency and rigor of the sampling strategy.

3. *The data integration strategy in the mixed methods design (e.g., triangulation or complementarity) needs explicit description in Methods or Discussion.*

Thank you for this helpful suggestion. We have revised the Methods and Discussion sections to explicitly describe our data integration strategy within the mixed methods design.

As described in our revised manuscript, our study employed a convergent mixed methods design, in which quantitative and qualitative data were collected concurrently, analyzed separately, and then integrated during interpretation. Integration occurred through a multi-stage coding process that combined inductive thematic analysis (Stages 1 and 2) with deductive coding frameworks (Stages 3 and 4). We have clarified that our integration strategy reflects complementarity rather than triangulation. Specifically, qualitative findings were used to contextualize and explain quantitative patterns (e.g., barriers such as fear of falling and transportation challenges help interpret low PA levels). This approach allowed us to capture both measurable activity patterns and the lived experiences underlying them (see Methods, pages 9-10, lines 199-207; 216-218; Fig 2; Discussion, page 37, lines 857-862).

4. *Theoretical Integration: While the Social Ecological Model is cited, its operationalization is inconsistent. Authors should map each theme and subtheme directly to model domains (intrapersonal, interpersonal, community, policy) to strengthen conceptual rigor.*

We appreciate this suggestion. To strengthen conceptual rigor, we revised Table 4 (page 37, line 871) to explicitly map each theme and subtheme to the corresponding SEM domain. For example, intrapersonal factors included fear of falling, comorbidities, and adaptive strategies; interpersonal factors encompassed social support and caregiving dynamics; organizational factors involved healthcare and community programs; community-level factors reflected neighborhood accessibility and transportation; and policy-level factors related to infrastructure and resource allocation. As shown in Fig 3, this mapping underscores the multi-level nature of barriers and facilitators and illustrates how ecological domains intersect to shape physical activity engagement.

5. *The Discussion should elaborate how findings inform or extend this framework in disability and diabetes contexts.*

Thank you for this important point. We have revised the Discussion section to elaborate on how our findings inform and extend the Social Ecological Model (SEM) in the context of disability and diabetes. Specifically, we highlight how our results demonstrate unique interactions between intrapersonal, interpersonal, organizational, community, and policy-level factors for individuals with vision loss and type 2 diabetes. As described in the revised manuscript, this work extends the SEM by illustrating the compounded barriers and facilitators that arise from co-occurring chronic conditions and disability (e.g., pages 40-41, lines 929-940).

6. *The Discussion focuses on descriptive barriers/facilitators but lacks connection to multidisciplinary rehabilitation, adaptive exercise, or policy implications. Integrating previous evidence could emphasize how structured physical activity frameworks can bridge psychosocial and physical challenges in chronic disability contexts. Add the phrase: “Previous research has emphasized the rehabilitative and preventive value of structured physical activity programs in promoting both mental and physical well-being among individuals with chronic disabilities [Nasif et al., 2025,* [*https://doi.org/10.31579/2578-8868/359*](https://nam11.safelinks.protection.outlook.com/?url=https%3A%2F%2Fdoi.org%2F10.31579%2F2578-8868%2F359&data=05%7C02%7Cemily.nicklett%40utsa.edu%7Cb3eb2dbda78b4c9f6c7f08de22624488%7C3a228dfbc64744cb88357b20617fc906%7C0%7C1%7C638986002641982185%7CUnknown%7CTWFpbGZsb3d8eyJFbXB0eU1hcGkiOnRydWUsIlYiOiIwLjAuMDAwMCIsIlAiOiJXaW4zMiIsIkFOIjoiTWFpbCIsIldUIjoyfQ%3D%3D%7C0%7C%7C%7C&sdata=kv1dMZ4vS6ZZU8NvmcE9nizNzxtr4Jx4jmDQu3HtCb4%3D&reserved=0) *]. Building on this perspective, our study helps extend this framework by identifying context-specific barriers and facilitators that can inform adaptive exercise models and inclusive rehabilitation strategies for adults with visual impairment and type 2 diabetes.”*

We appreciate this valuable suggestion and have incorporated this feedback into our revisions. Specifically, we developed a new figure (Fig 4) that highlights key implications for practice and policy, including: (a) Rehabilitation and adaptive exercise; (b) Social and behavioral supports; (c) Organizational and community design; and (d) Policy and infrastructure. These additions build upon the expanded discussion noted in Comment #5, which now emphasizes connections to multidisciplinary rehabilitation and adaptive exercise frameworks. While we considered integrating the suggested text verbatim, we believe the revised discussion and the new figure collectively address the Reviewer’s concerns by situating our findings within broader rehabilitative and policy contexts.

Reviewer 2:

1. *Explain the method used for random sampling within the recruitment pool to ensure representativeness and reduce selection bias.*

Thank you for this important point. We have clarified the random sampling procedure in the revised Methods section:

“To achieve a sample of 30 participants (this number was selected to enable adequately powered bivariate comparisons), contact information was provided for 200 randomly selected clients (Fig 1). To minimize selection bias and ensure representativeness of the Vibrant Works client population with T2D and vision loss, we used a simple random sampling approach. This method ensured that every eligible client had an equal probability of being contacted, reducing systematic bias and supporting representativeness of the organizational population. After excluding clients without contact information (n=96), we generated a revised sampling frame of 2,165 eligible clients. From this frame, we used a computer-generated random number sequence to select clients for recruitment in batches of 25, until we reached our target of 30 participants. In total, 200 clients were contacted, of whom 180 were eligible (20 were ineligible due to being deceased or for not meeting vision or diabetes status criteria), resulting in a response rate of 16.67% (Fig 1)” (page 7, lines 134-145; Fig 1).

2. *Examine the consequences of the low quantitative response rate and its potential impact on generalizability.*

We appreciate this important suggestion. We have added language to the Limitations section to explicitly address the implications of the low response rate:

“The response rate was 16.7% (30 of 180 eligible participants), which introduces potential non-response bias and limits generalizability. Individuals who agreed to participate may differ systematically from those who declined or were unreachable, for example, in health status, functional ability, or access to resources. As a result, findings may overrepresent individuals who are more engaged with community services, have higher levels of motivation to discuss diabetes self-management, or are generally more able and willing to participate in research. While the sample reflects the demographic composition of the eligible organizational client population, caution should be exercised in extrapolating results to broader populations of adults with T2D and vision loss, particularly those not connected to community-based organizations” (page 43, lines 983-991).

3. *Provide reliability statistics such as Cronbach’s α for the Exercise Behaviors Scale within this particular group or reference previous validation studies.*

Thank you for this suggestion to include reliability statistics for the Exercise Behaviors Scale from prior and current studies.

Psychometrics from prior studies: We have added information on reliability for the Exercise Behaviors Scale and the Chronic Disease Self-Management Program (CDSMP) measurement set. We now note in our revised manuscript that the Exercise Behaviors Scale, as a unique set, has not been psychometrically validated in prior research:

“While prior studies have found high internal consistency for related CDSMP scales, such as self-efficacy (Cronbach’s α ≈ .91; Lorig et al., 2001), no published factor-analytic studies have confirmed dimensionality of the Exercise Behaviors Scale specifically or assessed measurement invariance across populations” (page 42, lines 969-976).

While we recognize the importance of reliability metrics, in our study (n=30), we have opted not to include Cronbach’s α in the manuscript for the following reasons:

(1) The Exercise Behaviors Scale was designed as a pragmatic tool to estimate weekly physical activity minutes rather than as a psychometric measure of a latent construct.
(2) Our sample size (n=30) is small, and several items had very low variance, which would produce unstable α estimates and wide confidence intervals.
(3) Reporting such estimates could be misleading without proper context or validation studies.

Instead, we reference prior work demonstrating strong internal consistency for related CDSMP scales (Cronbach’s α ≈ .91; Lorig et al., 2001) and note in the Limitations section that future research should examine the reliability and validity of the Exercise Behaviors Scale in larger, diverse samples:

“Further studies should validate the subscale in larger, more diverse samples, including reliability testing and confirmatory factor analysis, to strengthen its use as a standalone instrument” (page 42, lines 974-976).

4. *Explicitly explain the approach taken to address any missing data during the statistical analyses.*

Thank you for this comment. We confirm that there were no missing data for any variables included in the quantitative analyses. All participants completed the included closed-ended questionnaire in full, and all responses were successfully recorded and entered into the dataset. Therefore, no imputation or special handling of missing data was required.

5. *Provide precise p-values, confidence intervals, and effect sizes for major quantitative comparisons to enhance reporting clarity.*

We appreciate this important suggestion. We have revised Table 3 to include precise p-values, confidence intervals, and effect sizes (i.e., ANOVA coefficients or odds ratios) for all comparisons to enhance reporting clarity (pages 15-16, lines 271-275). We have updated the Methods (pages 10-11, lines 219-229) and Results (page 14, lines 256-270) sections to reflect this revised approach.

6. *Think about including a short description or table outlining the qualitative coding framework or key themes as additional content.*

Thank you for this helpful suggestion. The coding framework is identified in **S1 Table: Qualitative Coding Scheme and Process**, and **Table 4** now presents the final themes organized by social-ecological level. We believe this addresses the request for clarity and additional context.

7. *Streamline redundant text in the Results section and emphasize main conclusions instead of reiterating table figures.*

Thank you for this helpful suggestion. We have streamlined the Results section by reducing redundant text and focusing on key findings rather than repeating table values. These revisions improve clarity and emphasize the main conclusions as recommended (page 14, lines 257-270).

8. *Broaden the conversation to link results with actionable approaches for rehabilitation after ICU stays or chronic illnesses and accessible physical activity initiatives.*

We appreciate this important suggestion. We have expanded the Discussion to explicitly connect our findings to rehabilitation contexts and accessible physical activity initiatives. Specifically, we added language highlighting how strategies identified in the study (such as modular “little-by-little” routines, caregiver-supported navigation, and adaptive equipment) can inform hospital-to-home transitions and chronic disease management or rehabilitation programs. We also note the relevance of community-based resources (e.g., senior centers, peer-led activity groups) and scalable approaches such as home-based exercise programs and telehealth-supported physical therapy. These additions appear in the Discussion sections (pages 41-42, lines 954-962) and in a new figure (Fig 4).

9. *More explicitly recognize the potential for self-report bias and the limitations associated with sample size and recruitment at a single site.*

Thank you for this important observation. We have strengthened the Limitations section to explicitly acknowledge these issues. Specifically, we added language noting that physical activity was assessed using self-reported measures, which may introduce recall and social desirability bias. We also emphasized that the small sample size limited statistical power and produced wide confidence intervals for some subgroups. Finally, we clarified that recruitment from a single community-based organization in one urban area may limit generalizability to individuals with T2D and vision loss who are not engaged with similar services or who reside in different geographic or cultural contexts. These revisions appear in the Limitations section of the Discussion (pages 42-43, lines 976-990).

10. *Enhance the data availability statement to comply with PLOS ONE guidelines by placing the de-identified dataset and codebook in a public repository.*

Thank you for this comment. Unfortunately, we are unable to share the dataset publicly or on request due to restrictions imposed by the University of Texas at San Antonio Institutional Review Board (IRB). The IRB determined that sharing even a de-identified dataset would conflict with the consent agreement provided to participants, which stated that data would only be accessed by the study team and destroyed three years after the study. This restriction was implemented to protect a geographically specific and potentially vulnerable population.

To comply with *PLOS ONE* guidelines as fully as possible under these constraints, we have updated the Data Availability Statement to clarify these IRB restrictions and provide contact information for the UTSA IRB for any inquiries. We have also ensured that all relevant summary data are presented in the manuscript tables and figures to maximize transparency.

11. *Verify that all figures and tables are consistent, correctly labeled, and formatted to publication standards.*

Thank you for this comment. We have carefully reviewed all tables and figures to ensure consistency in labeling, numbering, and formatting according to *PLOS ONE* guidelines. Captions have been standardized, abbreviations clarified, and table structures aligned with journal requirements. All references to tables and figures in the text have been verified for accuracy. These updates appear throughout the manuscript.

12. *Perform a thorough review of the language and grammar to fix minor punctuation and wording mistakes*.

Thank you for this comment. We have conducted a comprehensive review of the manuscript to correct minor punctuation, grammar, and wording issues. Edits were made throughout the text to improve clarity, readability, and adherence to journal style guidelines. The revisions appear across the Introduction, Methods, Results, and Discussion Sections.

13. *Incorporate information regarding the regional or national prevalence of vision loss due to diabetes to improve the introduction’s relevance to public health.*

### Thank you for this helpful suggestion. We have revised the Introduction to include both national and regional prevalence data. Specifically, we added the prevalence of vision-threatening DR in the United States (1.8 million). We also incorporated local data indicating that nearly 28% of people with diabetes in Bexar County show signs of diabetic retinopathy. We emphasized how these figures underscore the significant impact of vision loss due to diabetes both nationally and regionally (page 3, lines 43-53; page 43, lines 991-994).

14. *Conclude by highlighting how these insights can inform future extensive or long-term studies focused on accessible physical activity programs for individuals experiencing vision impairment.*

Thank you for this important suggestion. We integrated this context into the conclusion to emphasize the implications of our findings for policy and practice (page 43; lines 997-1001).

Reviewer 3:

*Major Comments*

1. *Data sharing: Consider depositing de-identified data in a controlled repository to comply with PLOS ONE’s data availability requirements*

Thank you very much for this comment. Unfortunately, we are unable to share the dataset publicly or on request due to restrictions by the University of Texas at San Antonio Institutional Review Board (IRB) and consent agreement. The IRB determined that sharing even a de-identified dataset would conflict with the consent agreement provided to participants, which stated that data would only be accessed by the study team and destroyed three years after the study. This restriction was implemented to protect a geographically specific and potentially vulnerable population. We have updated our data availability statement accordingly.

2. *Clarify integration: In the Methods, briefly describe how the quantitative and qualitative results were integrated (beyond “convergent inductive-deductive design”) – for example, how statistical trends informed thematic interpretation.*

We appreciate this suggestion. Our revised manuscript now describes the integration of qualitative and quantitative results in greater detail. We now describe how integration occurred through a multi-stage coding process that combined inductive thematic analysis (Stages 1 and 2) with deductive coding frameworks (Stages 3 and 4). We have clarified that our integration strategy reflects complementarity rather than triangulation. Specifically, qualitative findings were used to contextualize and explain quantitative patterns (e.g., barriers such as fear of falling and transportation challenges help interpret low PA levels). This approach allowed us to capture both measurable activity patterns and the lived experiences underlying them (see Methods, pages 9-10, lines 199-207, 216-218 Fig 2; S1 Table; Discussion, page 37, lines 857-862).

3. *Participant recruitment clarity: The description of inclusion/exclusion and random selection could be summarized in the text and expanded in a supplementary figure to improve readability.*

Thank you for this helpful suggestion. We have incorporated your recommendation, along with related feedback from Reviewer 1, to improve clarity and readability of the sampling strategy. Specifically:

- Summary in Text: We revised the *Participant Recruitment* section to provide a concise description of the inclusion/exclusion criteria (127-132) and random selection (134-145) processes.
- Expand Detail in Supplementary Figure and Additional Clarifications: We included a figure (Fig 1) highlighting key information about the sampling flow, including the initial sampling frame, exclusions, random selection steps, and participant enrollment.
- Per the suggestion from Reviewer 1, we also added details on recruitment channels, justification for inclusion/exclusion criteria, representativeness of blind versus low-vision participants, and rationale for sample size. These revisions strengthen transparency and rigor in describing the sampling strategy (pages 6-7, lines 124-148).

We appreciate this important feedback, which has helped us enhance both clarity and accessibility of this section.
