# Supplementary material for: “You never know what’s in front of you”: A mixed methods study of barriers and facilitators to physical activity among blind and low-vision adults with type 2 diabetes
Source: PLoS One. 2026 Jun 17;21(6):e0332565. doi: 10.1371/journal.pone.0332565 (PMC13274851; doi:10.1371/journal.pone.0332565)
Supplement: Attachment — Submitted filename: Response to Reviewers r2 2.15.26.docx [file pone.0332565.s004.docx]

February 15, 2026

Maheshkumar Baladaniya, PT, MS, DPT
Academic Editor, *PLOS ONE*

Response to Reviewers

RE: “**You never know what’s in front of you”: A mixed methods study of barriers and facilitators to physical activity among blind and low vision adults with type 2 diabetes** (Submission ID: PONE-D-25-47274)

Dear Dr. Baladaniya,

Thank you for the opportunity to revise our manuscript. We have carefully revised the paper in response to the reviewers’ recommendations. We are grateful to you and the reviewers for the thoughtful, constructive feedback throughout the review process, resulting in a strengthened manuscript.

In the response below, we address each reviewer’s comment and indicate the corresponding line numbers where revisions can be found in the revised, unmarked version of the manuscript. All substantive edits are also visible in the marked-up version of the manuscript (“Revised Manuscript with Track Changes”), in accordance with editorial guidelines.

We appreciate the time and expertise invested by the editorial team and reviewers, and we thank you for considering this revised manuscript for publication in *PLOS ONE.*

Sincerely,

Emily J. Nicklett, PhD, MSW (Corresponding Author)

Associate Professor of Social Work and Public Health

College for Health, Community and Policy

University of Texas at San Antonio

Email: [Emily.Nicklett@utsa.edu](mailto:Emily.Nicklett@utsa.edu)

Editorial Comments:

1. *If the reviewer comments include a recommendation to cite specific previously published works, please review and evaluate these publications to determine whether they are relevant and should be cited. There is no requirement to cite these works unless the editor has indicated otherwise.*

We appreciate this guidance. No specific citations were recommended by the reviewers in this round of review.

2. *Please review your reference list to ensure that it is complete and correct. If you have cited papers that have been retracted, please include the rationale for doing so in the manuscript text, or remove these references and replace them with relevant current references. Any changes to the reference list should be mentioned in the rebuttal letter that accompanies your revised manuscript. If you need to cite a retracted article, indicate the article’s retracted status in the References list and also include a citation and full reference for the retraction notice.*

We carefully reviewed our reference list to ensure completeness and accuracy and corrected minor errors. None of the cited papers have been retracted. One article previously cited as a preprint (Lee et al., 2026) has since been formally published, and the final publication details have now been updated in the reference list. In addition, we added one new reference (Lincoln & Guba, 1985) in response to reviewer feedback (Reviewer 4, Comment 5), as discussed below.

Reviewer 1:

1. *All comments have been addressed. Authors had satisfactorily addressed all issues raised by the reviewer and are now ready for acceptance. It is ready for the next phase of the editorial process.*

We appreciate the feedback and find that the manuscript has been significantly strengthened due to the reviewer’s suggestions.

Reviewer 4:

1. *In the quantitative phase, please clarify and justify why only 30 participants were selected, especially given that many were eligible.*

Thank you for this comment. We have clarified this point in the *Participant Recruitment* section (lines 123-155). Although a larger number of individuals met eligibility criteria, enrollment was intentionally limited to 30 participants to align with the exploratory objectives of the quantitative phase and to ensure feasibility within the study’s resource and accessibility constraints. The quantitative component was designed to provide descriptive characteristics of the sample, characterize preliminary patterns, and support integration with qualitative findings rather than to serve as a fully powered hypothesis-testing analysis. The manuscript has been revised to reflect this rationale and to ensure appropriate interpretation of the quantitative results (lines 124-143; 983-987).

2. *Explain why systematic random sampling was chosen for participant selection in the quantitative phase.*

Thank you for this comment. We have clarified the sampling approach in the manuscript (line 139). Participant selection for the quantitative phase used simple random sampling, not systematic random sampling. Eligible clients were randomly selected from the organizational roster using a computer-generated random number sequence so that all eligible individuals had an equal probability of selection. The methods section has been revised to reflect this more precise terminology and to briefly explain the rationale for this approach (lines 136-147; Fig 1).

3. *With such a small sample size, are you confident that a Chi-square test is appropriate? It might be worth considering Fisher’s exact test depending on the data.*

We appreciate this suggestion. Given our small sample (N=30) and the presence of sparse/zero cell counts in several categorical predictors, we re-estimated all categorical associations using Fisher’s exact tests. Results and conclusions remained unchanged. Because Fisher’s exact test provides valid inferences for sparse contingency tables, we have updated the Methods (lines 242-247), Results (lines 282-287), Table 3 (line 288), and table footnotes (lines 291-294) to reflect this revision.

4. *For the qualitative phase, please clarify the language criteria for participants and the interview process. How were interviews conducted, and what were the interviewers’ language proficiencies? If non-English languages were used, describe the translation and transcription processes.*

Thank you for this comment. We have clarified the language criteria and interview procedures in the Methods section (lines 163-169). Participants were eligible to complete interviews in either English or Spanish, according to their language preference. All interviews were conducted by trained members of the study team; Spanish-language interviews were conducted by bilingual (English-Spanish) interviewers. The qualitative interview guide and quantitative instruments were translated into Spanish and verified by two bilingual members of the study team before data collection.

Interviews conducted in English and Spanish followed the same semi-structured protocol and were administered in a comparable manner. All interviews were audio-recorded, transcribed verbatim, and, for Spanish-language interviews, translated into English by two bilingual interviewers (trained in qualitative methods) who conducted the interviews. This approach ensured conceptual consistency across languages and minimized loss of meaning during translation. The manuscript has been revised to reflect these procedures (lines 157-169).

5. *Regarding study rigor and trustworthiness in the qualitative phase, please detail how you addressed criteria like transferability, credibility, dependability, and confirmability.*

Thank you for this comment. Drawing on Lincoln & Guba (1985), we have clarified procedures used to enhance rigor and trustworthiness in the qualitative phase, guided by established qualitative criteria (e.g., transferability, credibility, dependability, and confirmability).

Credibility was enhanced through co-development of the interview guide with the community partner, semi-structured interviews, investigator triangulation, piloting before implementation with members of the target population, and collaborative analysis with the community partner. Two researchers independently coded line-by-line coding of all transcripts and met regularly to discuss interpretations, resolve discrepancies, and reach consensus. Engagement with the community partner throughout the study design, data collection, and interpretation further strengthened credibility.

Dependability was addressed by using a systematic, multi-stage analytic process, detailed analytic memoing, and a maintained audit trail of analytic decisions. Coding decisions, analytic memos, and iterative refinements to the codebook were documented, enabling consistency in analytic procedures across transcripts.

Confirmability was enhanced through reflexive practices, double-coding, and carefully grounding interpretations in verbatim participant quotations. Researchers engaged in reflexive discussions to examine assumptions and potential biases, and analytic decisions were grounded in participants’ verbatim data. The use of double-coding and consensus meetings further reduced individual researcher bias.

Transferability was supported by providing rich, thick descriptions of the study context, participant characteristics, and analysis procedures, allowing readers to assess the applicability of findings to other settings or populations. While the study is not intended to produce statistically generalizable findings, detailed contextual information enables analytic transferability.

We incorporated an explicit discussion of these strategies in the Methods section (lines 223-232). We appreciate this important feedback, which has helped us enhance the rigor and transparency of the qualitative component.
