# Supplementary material for: “You never know what’s in front of you”: A mixed methods study of barriers and facilitators to physical activity among blind and low-vision adults with type 2 diabetes
Source: PLoS One. 2026 Jun 17;21(6):e0332565. doi: 10.1371/journal.pone.0332565 (PMC13274851; doi:10.1371/journal.pone.0332565)
Supplement: Attachment — Submitted filename: Response to Reviewers r3.docx [file pone.0332565.s005.docx]

April 20, 2026

Maheshkumar Baladaniya, PT, MS, DPT
Academic Editor, *PLOS ONE*

Response to Reviewers

RE: “**You never know what’s in front of you”: A mixed methods study of barriers and facilitators to physical activity among blind and low vision adults with type 2 diabetes** (Submission ID: PONE-D-25-47274)

Dear Dr. Baladaniya,

Thank you for the opportunity to revise our manuscript. We have carefully revised the paper in response to the reviewers’ recommendations. We are grateful for the constructive feedback, which has resulted in a more cohesive and methodologically transparent manuscript.

Key revisions in this version include:

- Statistical Harmonization: We now employ Cramér’s *V* effect sizes, a more mathematically appropriate measure of association, without the need for reference-group modeling.
- Framing and Consistency: We have removed conflicting claims regarding sample size “adequacy”. The manuscript now consistently frames N=30 as a limitation, while justifying the exploratory use of exact statistical methods. We ensured that the variables shown in Tables 2 and 3 consistently followed sentence case.
- Limitations: The discussion section has been expanded to explicitly acknowledge the trade-offs of our statistical choices, including the conservative nature of Fisher’s exact test and the sensitivity of omnibus effect sizes to table dimensions.

Below, we address the Reviewer comments and feedback, indicating the corresponding line numbers where revisions can be found in the unmarked version of the manuscript. All substantive edits are also visible in the marked-up version of the manuscript.

We appreciate the time and expertise invested by the editorial team and reviewers, and we thank you for considering this revised manuscript for publication in *PLOS ONE.*

Sincerely,

Emily J. Nicklett, PhD, MSW (Corresponding Author)

Associate Professor of Social Work and Public Health

College for Health, Community and Policy

University of Texas at San Antonio

Email: [Emily.Nicklett@utsa.edu](mailto:Emily.Nicklett@utsa.edu)

Editorial Comments:

1. *If the reviewer comments include a recommendation to cite specific previously published works, please review and evaluate these publications to determine whether they are relevant and should be cited. There is no requirement to cite these works unless the editor has indicated otherwise.*

We appreciate this guidance. No specific citations were recommended by the reviewers in this round of review.

2. *Please review your reference list to ensure that it is complete and correct. If you have cited papers that have been retracted, please include the rationale for doing so in the manuscript text, or remove these references and replace them with relevant current references. Any changes to the reference list should be mentioned in the rebuttal letter that accompanies your revised manuscript. If you need to cite a retracted article, indicate the article’s retracted status in the References list and also include a citation and full reference for the retraction notice.*

We carefully reviewed our reference list to ensure completeness and accuracy.

Reviewer 5:

1. *Your recruitment methods seem expertly done, and the demographics of the subjects is good. The closed-ended questionnaires appear appropriate, and ADI, Walk Score, and Transit Score all seem valid. The data processing and cross-checking methods appear robust. Table 4 is very helpful, and the theme/sub-themes are well-reasoned. Figure 4 is well-organized and provides useful recommendations for the intended audience of this work.*

We appreciate the positive feedback.

Reviewer 6:

1. *This is a meaningful mixed-methods study on physical activity among adults with type 2 diabetes and blindness/low vision. The manuscript has improved substantially through revision, particularly by clarifying recruitment procedures, interview protocols, qualitative rigor, and the overall presentation of the mixed-method design.*

We are grateful for these comments and agree that the manuscript has greatly improved through the revision process.

2. *My remaining concern is the statistical presentation in Table 3. The manuscript states that Fisher’s exact tests were used for categorical predictors of small cell counts, yet single odds ratios are reported for multi-category variables such as age groups and employment status. It is unclear how these ORs were derived, and the current presentation may confuse readers. The reporters should either report only omnibus Fisher’s exact p-values or reanalyze these variables using clearly specified logistic regression models with reference categories and category-specific ORs with 95% CIs.*

We thank the Reviewers for this insightful comment regarding the ambiguity of reporting a single Odds Ratio for multi-category variables (e.g., age group, race/ethnicity). We agree that a single OR in these instances lacks a clear reference point and can be mathematically misleading.

To resolve this and ensure statistical clarity, we have updated our presentation of effect sizes in Table 3 as follows:

a) For multicategory variables (>2 levels), we have replaced the single OR with Cramér’s *V*. This provides an appropriate omnibus measure of association that is not dependent on a reference category.
b) For binary and continuous variables, we have retained the Odds Ratio (OR) as the measure of association, as it remains the standard and most interpretable effect size for these data types.
c) Justification for Approach: While the Reviewer suggested Category-specific ORs via logistic regression, we opted for the omnibus Cramér’s *V* approach given our small sample size (N=30), as reference-group modeling with small cell counts can result in unstable estimates and “not estimable” parameters (as seen in some of our descriptive cells), whereas Cramér’s *V* remains a robust and informative indicator of effect magnitude (Agresti, 2013).

These changes are now reflected in the Methods section (lines 242-250), Table 3 and its associated Note (lines 292-299), and the updated Results for the bivariate analysis (lines 285-291), and the Discussion section (lines 988-998).

3. *A second issue is the wording around sample size. The manuscript describes the quantitative component as exploratory but also suggests that the sample size was adequate for bivariate comparisons, while the Discussion later acknowledges limited power. The language should be made more consistent.*

We appreciate the Reviewer’s call for consistency regarding our sample size description. We have revised the manuscript to remove conflicting claims of “adequacy” while maintaining that the analyses are exploratory. We now consistently frame the *N = 30* sample as a limitation that necessitated the use of exact statistical methods (Fisher’s exact tests) and omnibus effect sizes (Cramér’s *V*), which are methodologically appropriate for small-cell bivariate associations even when overall statistical power is limited. Additionally, we have expanded the Limitations section to explicitly acknowledge the inherent trade-offs of these methods, including the conservative nature of Fisher’s exact test and the sensitivity of Cramér’s *V* to table dimensions and empty cells. These changes are now reflected in the Methods (lines 136-137) and Discussion (lines 988-998) sections.

Reviewer 7:

*N/A – All comments have been addressed.*

We appreciate the positive feedback.

We are grateful to the anonymous Reviewers for their constructive feedback and insights.
