# Supplementary material for: “You never know what’s in front of you”: A mixed methods study of barriers and facilitators to physical activity among blind and low-vision adults with type 2 diabetes
Source: PLoS One. 2026 Jun 17;21(6):e0332565. doi: 10.1371/journal.pone.0332565 (PMC13274851; doi:10.1371/journal.pone.0332565)
Supplement: Peer review history

# Peer review: Decision Letter 0

**DOI:** 10.1371/journal.pone.0332565.r001

**Authors:** Maheshkumar Baladaniya

12 Nov 2025

PONE-D-25-47274

“You never know what’s in front of you”: A mixed methods study of barriers and facilitators to physical activity among blind and low-vision adults with type 2 diabetes

PLOS ONE

Dear Dr. Nicklett,

Thank you for submitting your manuscript to PLOS ONE. After careful consideration, we feel that it has merit but does not fully meet PLOS ONE’s publication criteria as it currently stands. Therefore, we invite you to submit a revised version of the manuscript that addresses the points raised during the review process.

Please submit your revised manuscript by Dec 27 2025 11:59PM. If you will need more time than this to complete your revisions, please reply to this message or contact the journal office at plosone@plos.org. When you're ready to submit your revision, log on to https://www.editorialmanager.com/pone/ and select the 'Submissions Needing Revision' folder to locate your manuscript file.

Please include the following items when submitting your revised manuscript:

- A rebuttal letter that responds to each point raised by the academic editor and reviewer(s). You should upload this letter as a separate file labeled 'Response to Reviewers'.
- A marked-up copy of your manuscript that highlights changes made to the original version. You should upload this as a separate file labeled 'Revised Manuscript with Track Changes'.
- An unmarked version of your revised paper without tracked changes. You should upload this as a separate file labeled 'Manuscript'.

-->If you would like to make changes to your financial disclosure, please include your updated statement in your cover letter. Guidelines for resubmitting your figure files are available below the reviewer comments at the end of this letter.

If applicable, we recommend that you deposit your laboratory protocols in protocols.io to enhance the reproducibility of your results. Protocols.io assigns your protocol its own identifier (DOI) so that it can be cited independently in the future. For instructions see: https://journals.plos.org/plosone/s/submission-guidelines#loc-laboratory-protocols. Additionally, PLOS ONE offers an option for publishing peer-reviewed Lab Protocol articles, which describe protocols hosted on protocols.io. Read more information on sharing protocols at https://plos.org/protocols?utm_medium=editorial-email&utm_source=authorletters&utm_campaign=protocols.

We look forward to receiving your revised manuscript.

Kind regards,

Maheshkumar Baladaniya

Academic Editor

PLOS ONE

Journal Requirements:

When submitting your revision, we need you to address these additional requirements.

1. Please ensure that your manuscript meets PLOS ONE's style requirements, including those for file naming. The PLOS ONE style templates can be found at

https://journals.plos.org/plosone/s/file?id=wjVg/PLOSOne_formatting_sample_main_body.pdf and

https://journals.plos.org/plosone/s/file?id=ba62/PLOSOne_formatting_sample_title_authors_affiliations.pdf

2. We note that the grant information you provided in the ‘Funding Information’ and ‘Financial Disclosure’ sections do not match.

When you resubmit, please ensure that you provide the correct grant numbers for the awards you received for your study in the ‘Funding Information’ section.

3. Thank you for stating the following in the Competing Interests section:

Several coauthors (TW, JJC) are employed by Vibrant Works, an organization mentioned in the manuscript. Additionally, collaborative grants involving Vibrant Works have been received by authors on this manuscript (TW, JJC, EJN). The remaining authors declare to have no conflicts of interest. This does not alter our adherence to PLOS One policies on sharing data and materials.

Please confirm that this does not alter your adherence to all PLOS ONE policies on sharing data and materials, by including the following statement: “This does not alter our adherence to PLOS ONE policies on sharing data and materials.” (as detailed online in our guide for authors http://journals.plos.org/plosone/s/competing-interests). If there are restrictions on sharing of data and/or materials, please state these. Please note that we cannot proceed with consideration of your article until this information has been declared.

Please include your updated Competing Interests statement in your cover letter; we will change the online submission form on your behalf.

4. Please include your full ethics statement in the ‘Methods’ section of your manuscript file. In your statement, please include the full name of the IRB or ethics committee who approved or waived your study, as well as whether or not you obtained informed written or verbal consent. If consent was waived for your study, please include this information in your statement as well.

5. We note that you have indicated that there are restrictions to data sharing for this study. For studies involving human research participant data or other sensitive data, we encourage authors to share de-identified or anonymized data. However, when data cannot be publicly shared for ethical reasons, we allow authors to make their data sets available upon request. For information on unacceptable data access restrictions, please see http://journals.plos.org/plosone/s/data-availability#loc-unacceptable-data-access-restrictions.

Before we proceed with your manuscript, please address the following prompts:

a) If there are ethical or legal restrictions on sharing a de-identified data set, please explain them in detail (e.g., data contain potentially identifying or sensitive patient information, data are owned by a third-party organization, etc.) and who has imposed them (e.g., a Research Ethics Committee or Institutional Review Board, etc.). Please also provide contact information for a data access committee, ethics committee, or other institutional body to which data requests may be sent.

b) If there are no restrictions, please upload the minimal anonymized data set necessary to replicate your study findings to a stable, public repository and provide us with the relevant URLs, DOIs, or accession numbers. Please see http://www.bmj.com/content/340/bmj.c181.long for guidelines on how to de-identify and prepare clinical data for publication. For a list of recommended repositories, please see https://journals.plos.org/plosone/s/recommended-repositories. You also have the option of uploading the data as Supporting Information files, but we would recommend depositing data directly to a data repository if possible.

Please update your Data Availability statement in the submission form accordingly.

6. Please remove all personal information, ensure that the data shared are in accordance with participant consent, and re-upload a fully anonymized data set.

Note: spreadsheet columns with personal information must be removed and not hidden as all hidden columns will appear in the published file.

Additional guidance on preparing raw data for publication can be found in our Data Policy (https://journals.plos.org/plosone/s/data-availability#loc-human-research-participant-data-and-other-sensitive-data) and in the following article: http://www.bmj.com/content/340/bmj.c181.long.

7. If the reviewer comments include a recommendation to cite specific previously published works, please review and evaluate these publications to determine whether they are relevant and should be cited. There is no requirement to cite these works unless the editor has indicated otherwise.

8. Please review your reference list to ensure that it is complete and correct. If you have cited papers that have been retracted, please include the rationale for doing so in the manuscript text, or remove these references and replace them with relevant current references. Any changes to the reference list should be mentioned in the rebuttal letter that accompanies your revised manuscript. If you need to cite a retracted article, indicate the article’s retracted status in the References list and also include a citation and full reference for the retraction notice.

Additional Editor Comments:

There are few areas that needs to be address mentioned by reviewers.

[Note: HTML markup is below. Please do not edit.]

Reviewers' comments:

Reviewer's Responses to Questions

**Comments to the Author**

1. Is the manuscript technically sound, and do the data support the conclusions?

The manuscript must describe a technically sound piece of scientific research with data that supports the conclusions. Experiments must have been conducted rigorously, with appropriate controls, replication, and sample sizes. The conclusions must be drawn appropriately based on the data presented.

Reviewer #1: Yes

Reviewer #2: Yes

Reviewer #3: Yes

**********

2. Has the statistical analysis been performed appropriately and rigorously?

Reviewer #1: Yes

Reviewer #2: Yes

Reviewer #3: Yes

**********

3. Have the authors made all data underlying the findings in their manuscript fully available?

The PLOS Data policy requires authors to make all data underlying the findings described in their manuscript fully available without restriction, with rare exception (please refer to the Data Availability Statement in the manuscript PDF file). The data should be provided as part of the manuscript or its supporting information, or deposited to a public repository. For example, in addition to summary statistics, the data points behind means, medians and variance measures should be available. If there are restrictions on publicly sharing data—e.g. participant privacy or use of data from a third party—those must be specified.

Reviewer #1: Yes

Reviewer #2: No

Reviewer #3: Yes

**********

4. Is the manuscript presented in an intelligible fashion and written in standard English?

PLOS ONE does not copyedit accepted manuscripts, so the language in submitted articles must be clear, correct, and unambiguous. Any typographical or grammatical errors should be corrected at revision, so please note any specific errors here.

Reviewer #1: Yes

Reviewer #2: Yes

Reviewer #3: Yes

**********

5. Review Comments to the Author

Please use the space provided to explain your answers to the questions above. You may also include additional comments for the author, including concerns about dual publication, research ethics, or publication ethics. (Please upload your review as an attachment if it exceeds 20,000 characters)

Reviewer #1: The study adopts a mixed-methods approach grounded in the Social Ecological Model, contributing valuable insights for inclusive diabetes care and physical activity promotion. However, certain methodological, interpretive, and theoretical aspects require clarification and refinement to strengthen rigor, reproducibility, and conceptual linkage.

Major Issues

The quantitative component (Exercise Behaviors Scale) requires more detail: scoring, validity, and how results informed the qualitative phase.

Sampling strategy is insufficiently justified — the paper should clarify recruitment channels, inclusion/exclusion criteria, and representativeness of blind vs. low-vision participants.

Data integration strategy in the mixed-methods design (e.g., triangulation or complementarity) needs explicit description in Methods or Discussion.

Theoretical Integration

While the Social Ecological Model is cited, its operationalization is inconsistent. Authors should map each theme and subtheme directly to model domains (intrapersonal, interpersonal, community, policy) to strengthen conceptual rigor.

Discussion should elaborate how findings inform or extend this framework in disability and diabetes contexts.

The discussion focuses on descriptive barriers/facilitators but lacks connection to multidisciplinary rehabilitation, adaptive exercise, or policy implications. Integrating previous evidence could emphasize how structured physical activity frameworks can bridge psychosocial and physical challenges in chronic disability contexts. Add below phrase ….

“Previous research has emphasized the rehabilitative and preventive value of structured physical activity programs in promoting both mental and physical well-being among individuals with chronic disabilities [Nasif et al., 2025, https://doi.org/10.31579/2578-8868/359 ]. Building on this perspective, our study helps extend this framework by identifying context-specific barriers and facilitators that can inform adaptive exercise models and inclusive rehabilitation strategies for adults with visual impairment and type 2 diabetes.”

Reviewer #2: Explain the method used for random sampling within the recruitment pool to ensure representativeness and reduce selection bias.

Examine the consequences of the low quantitative response rate and its potential impact on generalizability.

Provide reliability statistics such as Cronbach’s α, for the Exercise Behaviors Scale within this particular group or reference previous validation studies.

Explicitly explain the approach taken to address any missing data during the statistical analyses.

Provide precise p-values, confidence intervals, and effect sizes for major quantitative comparisons to enhance reporting clarity.

Think about including a short description or table outlining the qualitative coding framework or key themes as additional content.

Streamline redundant text in the Results section and emphasize main conclusions instead of reiterating table figures.

Broaden the conversation to link results with actionable approaches for rehabilitation after ICU stays or chronic illnesses and accessible physical activity initiatives.

More explicitly recognize the potential for self-report bias and the limitations associated with sample size and recruitment at a single site.

Enhance the data availability statement to comply with PLOS ONE guidelines by placing the de-identified dataset and codebook in a public repository.

Verify that all figures and tables are consistent, correctly labeled, and formatted to publication standards.

Perform a thorough review of the language and grammar to fix minor punctuation and wording mistakes.

Incorporate information regarding the regional or national prevalence of vision loss due to diabetes to improve the introduction's relevance to public health.

Conclude by highlighting how these insights can inform future extensive or long-term studies focused on accessible physical activity programs for individuals experiencing vision impairment.

Reviewer #3: Overall Evaluation:

This is an important and original contribution addressing an under-researched intersection between diabetes self-management and vision impairment. The mixed-methods design offers a comprehensive understanding of the multilevel factors influencing PA participation. The study’s practical implications for community and policy interventions are well articulated. The collaboration with a community organization (Vibrant Works) enhances ecological validity and demonstrates strong stakeholder engagement.

Major Comments

Data sharing:

Consider depositing de-identified data (quantitative dataset and coded qualitative excerpts) in a controlled repository to comply with PLOS ONE’s data-availability requirements.

Clarify integration:

In the Methods, briefly describe how the quantitative and qualitative results were integrated (beyond “convergent inductive–deductive design”)—for example, how statistical trends informed thematic interpretation.

Participant recruitment clarity:

The description of inclusion/exclusion and random selection could be summarized in the text and expanded in a supplementary figure to improve readability.

**********

6. PLOS authors have the option to publish the peer review history of their article (what does this mean?). If published, this will include your full peer review and any attached files.

If you choose “no”, your identity will remain anonymous but your review may still be made public.

**Do you want your identity to be public for this peer review?**   For information about this choice, including consent withdrawal, please see our Privacy Policy.

Reviewer #1: No

Reviewer #2: No

Reviewer #3: **Yes:**  Elabbass Ali Abdelmahmuod

**********

[NOTE: If reviewer comments were submitted as an attachment file, they will be attached to this email and accessible via the submission site. Please log into your account, locate the manuscript record, and check for the action link “View Attachments”. If this link does not appear, there are no attachment files.]

To ensure your figures meet our technical requirements, please review our figure guidelines: https://journals.plos.org/plosone/s/figures

You may also use PLOS’s free figure tool, NAAS, to help you prepare publication quality figures: https://journals.plos.org/plosone/s/figures#loc-tools-for-figure-preparation.

NAAS will assess whether your figures meet our technical requirements by comparing each figure against our figure specifications.

Attachment Submitted filename: Comments.docx

# Author comment: Author response to Decision Letter 1

**DOI:** 10.1371/journal.pone.0332565.r002

16 Dec 2025

December 16, 2025

Maheshkumar Baladaniya, PT, MS, DPT

Academic Editor, PLOS ONE

Response to Reviewers

RE: “You never know what’s in front of you”: A mixed methods study of barriers and facilitators to physical activity among blind and low vision adults with type 2 diabetes (Submission ID: PONE-D-25-47274)

Dear Dr. Maheshkumar Baladaniya,

The manuscript has been revised in accordance with the anonymous reviewers’ suggestions. I appreciate the editor’s and reviewers’ thoughtful guidance and expertise on the manuscript. I have endeavored to be forthcoming and cooperative in addressing this constructive feedback and preparing the revised version of the manuscript.

Below, I include the suggestions from each reviewer and include the page number and lines where corresponding revisions are located in the revised, unmarked version of the manuscript. Changes are shown in the marked-up version (‘Revised Manuscript with Track Changes’), per editorial instructions. Revisions have also been made based on editorial instructions.

Thank you for the opportunity to revise this manuscript, which is being considered for publication in PLOS ONE.

Sincerely,

Emily J. Nicklett, PhD, MSW (Corresponding Author)

Associate Professor of Social Work and Public Health

College for Health, Community and Policy

University of Texas at San Antonio

Email: Emily.Nicklett@utsa.edu

-------

Editorial Comments:

1. Please ensure that the manuscript meets PLOS ONE’s style requirements, including those for file naming.

Thank you for this suggestion. We have reviewed the documents and made minor changes to align with the style requirements, including:

- The filename for S1 Table has been changed to S1_Table.

- The Figure names now include “.tiff” as part of the name (e.g., Fig1 changed to Fig1.tiff).

- Line numbers are now included in the title page.

- The corresponding author’s email address is no longer underlined/hyperlinked and now aligns with the style requirements.

2. The grant information provided in the ‘Funding Information’ and ‘Financial Disclosure’ Sections do not match. Please ensure that you provide the correct grant numbers for the awards you received for your study in the ‘Funding Information’ section.

The funding information is now consistent, and the discrepancies have been removed.

3. In the Competing Interests section, please confirm that the stated competing interests does not alter your adherence to all PLOS ONE policies on sharing data and materials, by including the following statement: “This does not alter our adherence to PLOS ONE policies on sharing data and materials. If there are restrictions on the sharing of data and/or materials, please state these. Please include this in your cover letter and it will be changed in the online submission form on your behalf.

We confirm that the competing interests do not alter adherence to PLOS ONE policies on sharing data and materials. We also identify our restrictions on data sharing. In response to this request, we include this information in our cover letter, with the request that it be changed in the online submission form on our behalf.

4. Please include your full ethics statement in the ‘Methods’ section of your manuscript file. In your statement, please include the full name of the IRB or ethics committee who approved or waived your study, as well as whether or not you obtained informed written or verbal consent. If consent was waived for your study, please include this information in your statement as well.

This information is included in the ‘Participant recruitment’ section of the ‘Methods’ section of the manuscript (page 7, lines 148-153).

5. We note that you have indicated that there are restrictions to data sharing for this study. For studies involving human participant data or other sensitive data, we encourage authors to share de-identified or anonymized data. However, when data cannot be publicly shared for ethical reasons, we allow authors to make their data sets available upon request. […] Before we proceed, please address the following prompts:

(a) If there are any ethical or legal restrictions on sharing a de-identified data set, please explain them in detail (e.g., data contain potentially identifying or sensitive patient information, data are owned by a third-party organization, etc.) and who has imposed them (e.g., a Research Ethics Committee or Institutional Review Board, etc.). Please also provide contact information for a data access committee, ethics committee, or other institutional body to which data requests may be sent.

The Institutional Review Board at the University of Texas at San Antonio has restricted sharing data, even if de-identified, publicly or upon request. Per the UTSA IRB (irb@utsa.edu), data sharing conflicts with the agreement provided to participants in the consent form for the study:

“Data will be kept in a secure, password-protected database and will only be accessed by members of the study team. Data will be kept for 3 years after the study is complete and will be destroyed after that time.”

The rationale for this approach was that it contains potentially identifying information for a geographically specific and potentially vulnerable study population, and that added protections were therefore needed.

Our COI statement now includes the following statement:

“In accordance with the University of Texas at San Antonio Institutional Review Board (irb@utsa.edu), and the terms of participant consent, the qualitative and quantitative datasets underlying this study cannot be shared publicly, as doing so would risk breaching participant confidentiality. Summary data and results are provided within the manuscript.”

(b) If there are no restrictions, please upload the minimal anonymized data set necessary to replicate your study findings to a stable, public repository and provide us with the relevant URLs, DOIs, or accession numbers.

Please update your Data Availability statement in the submission form accordingly.

The Data Availability statement has been updated accordingly.

6. Please remove all personal information, ensure that the data shared are in accordance with participant consent, and re-upload a fully anonymized data set.

We appreciate this suggestion. As the exact ages of participants included in the manuscript constitute identifying information, we have replaced the exact ages in Table 1 and throughout the manuscript with age ranges: 40-54, 55-64, 65-74, and 75+ and removed the ‘Household composition’ column. As we are not permitted to publish the data set (per University of Texas at San Antonio IRB) due to potential violation of participant consent processes, we unfortunately cannot upload or provide an anonymous dataset beyond what is shared in Table 1.

7. If the reviewer comments include a recommendation to cite specific previously published works, please review and evaluate these publications to determine whether they are relevant and should be cited. There is no requirement to cite these works unless the editor has indicated otherwise.

We appreciate this suggestion and proceeded accordingly.

8. Please review your reference list to ensure that it is complete and correct.

We have reviewed our reference list, made corrections, and confirm that it is complete and correct.

-------

Reviewer 1:

Major Issues

1. The quantitative component (Exercise Behaviors Scale) requires more detail: scoring, validity, and how results informed the qualitative phase

We appreciate this helpful suggestion. The ‘Data collection and procedures’ and ‘Limitations’ sections provide additional detail about the scoring and validity of the Exercise Behaviors Scale (page 8, lines 163-179; page 42, lines 969-976).

In these sections, we identify the scale part of the Chronic Disease Self-Management Program (CDSMP). The scale is widely used in CDSMP evaluations and research (e.g., CDC, 2013; Wilmoth et al., 2020). While the exercise behaviors-specific subscale has not been formally validated, internal consistency for other subscales in the CDSMP (e.g., self-efficacy) was high (Cronbach’s α ≈ .91). For the overall CDSMP, psychometric testing was conducted on a sample of 605 adults with chronic conditions, demonstrating acceptable reliability and responsiveness to change. The exercise behavior items specifically showed significant improvements at 4-6 months and 9-12 months post-intervention, supporting construct validity (Lorig et al. 2001). Subsequent meta-analyses and program evaluations have confirmed the sensitivity of these items to behavioral change across diverse populations, including older adults and people with disabilities, although no standalone factor analysis validation of the subscale has been published.

We revised the ‘Data analysis’ section to more clearly discuss how the Exercise Behaviors Scale informed that qualitative phase (pages 10-11, lines 216-229; Fig 2: Stage 4; S1 Table: Stage 4).

2. The sampling strategy is insufficiently justified—the paper should clarify recruitment channels, inclusion/exclusion criteria, and representativeness of blind vs. low-vision participants.

Thank you for this valuable feedback. We have revised the manuscript to provide greater clarity and justification for the sampling strategy (pages 6-7, lines 124-153; Fig 1):

(a) Recruitment Channels

We now explicitly state that participants were recruited through Vibrant Works, a nonprofit organization serving individuals who are blind or vision impaired. Recruitment relied on client records maintained by Vibrant Works, which include vision and diabetes status, ensuring access to the target population. Participants were contacted via phone using information provided by the organization.

(b) Inclusion and Exclusion Criteria

We have clarified that the inclusion criteria were (a) diagnosis of type 2 diabetes (T2D), (b) blindness or vision impairment documented by Vibrant Works, and (c) ability to provide verbal consent. Exclusion criteria included: (a) no diagnosis of T2D or blindness/vision impairment, or (b) inability to provide verbal consent due to language or health limitations. These criteria were selected to ensure participants could meaningfully engage in the study.

(c) Representative of Blind vs. Low-Vision Participants

We have added a statement including that the sample reflects the composition of Vibrant Works’ client population, which includes individuals with varying degrees of vision impairment. Specifically, of the 30 participants, 10 were blind and 20 had low vision, consistent with organizational records. This clarification addresses representativeness within the study population.

(d) Justification of Sample Size

We have included a rationale for the target sample size of 30 participants, noting that the number was selected to enable adequately powered bivariate comparisons while considering feasibility given recruitment challenges in this population.

These revisions appear in the ‘Participant Recruitment’ section of the manuscript (pages 6-7, lines 124-153). We believe these changes address the reviewer’s concerns and strengthen the transparency and rigor of the sampling strategy.

3. The data integration strategy in the mixed methods design (e.g., triangulation or complementarity) needs explicit description in Methods or Discussion.

Thank you for this helpful suggestion. We have revised the Methods and Discussion sections to explicitly describe our data integration strategy within the mixed methods design.

As described in our revised manuscript, our study employed a convergent mixed methods design, in which quantitative and qualitative data were collected concurrently, analyzed separately, and then integrated during interpretation. Integration occurred through a multi-stage coding process that combined inductive thematic analysis (Stages 1 and 2) with deductive coding frameworks (Stages 3 and 4). We have clarified that our integration strategy reflects complementarity rather than triangulation. Specifically, qualitative findings were used to contextualize and explain quantitative patterns (e.g., barriers such as fear of falling and transportation challenges help interpret low PA levels). This approach allowed us to capture both measurable activity patterns and the lived experiences underlying them (see Methods, pages 9-10, lines 199-207; 216-218; Fig 2; Discussion, page 37, lines 857-862).

4. Theoretical Integration: While the Social Ecological Model is cited, its operationalization is inconsistent. Authors should map each theme and subtheme directly to model domains (intrapersonal, interpersonal, community, policy) to strengthen conceptual rigor.

We appreciate this suggestion. To strengthen conceptual rigor, we revised Table 4 (page 37, line 871) to explicitly map each theme and subtheme to the corresponding SEM domain. For example, intrapersonal factors included fear of falling, comorbidities, and adaptive strategies; interpersonal factors encompassed social support and caregiving dynamics; organizational factors involved healthcare and community programs; community-level factors reflected neighborhood accessibility and transportation; and policy-level factors related to infrastructure and resource allocation. As shown in Fig 3, this mapping underscores the multi-level nature of barriers and facilitators and illustrates how ecological domains intersect to shape physical activity engagement.

5. The Discussion should elaborate how findings inform or extend this framework in disability and diabetes contexts.

Thank you for this important point. We have revised the Discussion section to elaborate on how our findings inform and extend the Social Ecological Model (SEM) in the context of disability and diabetes. Specifically, we highlight how our results demonstrate unique interactions between intrapersonal, interpersonal, organizational, community, and policy-level factors for individuals with vision loss and type 2 diabetes. As described in the revised manuscript, this work extends the SEM by illustrating the compounded barriers and facilitators that arise from co-occurring chronic conditions and disability (e.g., pages 40-41, lines 929-940).

6. The Discussion focuses on descriptive barriers/facilitators but lacks connection to multidisciplinary rehabilitation, adaptive exercise, or policy implications. Integrating previous evidence could emphasize how structured physical activity frameworks can bridge psychosocial and physical challenges in chronic disability contexts. Add the phrase: “Previous research has emphasized the rehabilitative and preventive value of structured physical activity programs in promoting both mental and physical well-being among individuals with chronic disabilities [Nasif et al., 2025, https://doi.org/10.31579/2578-8868/359 ]. Building on this perspective, our study helps extend this framework by identifying context-specific barriers and facilitators that can inform adaptive exercise models and inclusive rehabilitation strategies for adults with visual impairment and type 2 diabetes.”

We appreciate this valuable suggestion and have incorporated this feedback into our revisions. Specifically, we developed a new figure (Fig 4) that highlights key implications for practice and policy, including: (a) Rehabilitation and adaptive exercise; (b) Social and behavioral supports; (c) Organizational and community design; and (d) Policy and infrastructure. These additions build upon the expanded discussion noted in Comment #5, which now emphasizes connections to multidisciplinary rehabilitation and adaptive exercise frameworks. While we considered integrating the suggested text verbatim, we believe the revised discussion and the new figure collectively address the Reviewer’s concerns by situating our findings within broader rehabilitative and policy contexts.

-------

Reviewer 2:

1. Explain the method used for random sampling within the recruitment pool to ensure representativeness and reduce selection bias.

Thank you for this important point. We have clarified the random sampling procedure in the revised Methods section:

“To achieve a sample of 30 participants (this number was selected to enable adequate

Attachment Submitted filename: Response to Reviewers 12.16.25.docx

# Peer review: Decision Letter 1

**DOI:** 10.1371/journal.pone.0332565.r003

**Authors:** Maheshkumar Baladaniya

28 Jan 2026

PONE-D-25-47274R1

“You never know what’s in front of you”: A mixed methods study of barriers and facilitators to physical activity among blind and low-vision adults with type 2 diabetes

PLOS One

Dear Dr. Nicklett,

Thank you for submitting your manuscript to PLOS ONE. After careful consideration, we feel that it has merit but does not fully meet PLOS ONE’s publication criteria as it currently stands. Therefore, we invite you to submit a revised version of the manuscript that addresses the points raised during the review process.

Please submit your revised manuscript by Mar 14 2026 11:59PM. If you will need more time than this to complete your revisions, please reply to this message or contact the journal office at plosone@plos.org. When you're ready to submit your revision, log on to https://www.editorialmanager.com/pone/ and select the 'Submissions Needing Revision' folder to locate your manuscript file.

Please include the following items when submitting your revised manuscript:

- A letter that responds to each point raised by the academic editor and reviewer(s). You should upload this letter as a separate file labeled 'Response to Reviewers'.
- A marked-up copy of your manuscript that highlights changes made to the original version. You should upload this as a separate file labeled 'Revised Manuscript with Track Changes'.
- An unmarked version of your revised paper without tracked changes. You should upload this as a separate file labeled 'Manuscript'.

-->If you would like to make changes to your financial disclosure, please include your updated statement in your cover letter. Guidelines for resubmitting your figure files are available below the reviewer comments at the end of this letter.

If applicable, we recommend that you deposit your laboratory protocols in protocols.io to enhance the reproducibility of your results. Protocols.io assigns your protocol its own identifier (DOI) so that it can be cited independently in the future. For instructions see: https://journals.plos.org/plosone/s/submission-guidelines#loc-laboratory-protocols. Additionally, PLOS ONE offers an option for publishing peer-reviewed Lab Protocol articles, which describe protocols hosted on protocols.io. Read more information on sharing protocols at https://plos.org/protocols?utm_medium=editorial-email&utm_source=authorletters&utm_campaign=protocols.

We look forward to receiving your revised manuscript.

Kind regards,

Maheshkumar Baladaniya

Academic Editor

PLOS One

Journal Requirements:

1. If the reviewer comments include a recommendation to cite specific previously published works, please review and evaluate these publications to determine whether they are relevant and should be cited. There is no requirement to cite these works unless the editor has indicated otherwise.

2. Please review your reference list to ensure that it is complete and correct. If you have cited papers that have been retracted, please include the rationale for doing so in the manuscript text, or remove these references and replace them with relevant current references. Any changes to the reference list should be mentioned in the rebuttal letter that accompanies your revised manuscript. If you need to cite a retracted article, indicate the article’s retracted status in the References list and also include a citation and full reference for the retraction notice.

Additional Editor Comments :

There are still minor changes required to make it publishable. As described by the reviewer.

[Note: HTML markup is below. Please do not edit.]

Reviewers' comments:

Reviewer's Responses to Questions

**Comments to the Author**

1. If the authors have adequately addressed your comments raised in a previous round of review and you feel that this manuscript is now acceptable for publication, you may indicate that here to bypass the “Comments to the Author” section, enter your conflict of interest statement in the “Confidential to Editor” section, and submit your “Accept” recommendation.

Reviewer #1: All comments have been addressed

Reviewer #4: (No Response)

**********

2. Is the manuscript technically sound, and do the data support the conclusions?

The manuscript must describe a technically sound piece of scientific research with data that supports the conclusions. Experiments must have been conducted rigorously, with appropriate controls, replication, and sample sizes. The conclusions must be drawn appropriately based on the data presented.

Reviewer #1: Yes

Reviewer #4: Yes

**********

3. Has the statistical analysis been performed appropriately and rigorously?

Reviewer #1: Yes

Reviewer #4: No

**********

4. Have the authors made all data underlying the findings in their manuscript fully available?

The PLOS Data policy requires authors to make all data underlying the findings described in their manuscript fully available without restriction, with rare exception (please refer to the Data Availability Statement in the manuscript PDF file). The data should be provided as part of the manuscript or its supporting information, or deposited to a public repository. For example, in addition to summary statistics, the data points behind means, medians and variance measures should be available. If there are restrictions on publicly sharing data—e.g. participant privacy or use of data from a third party—those must be specified.

Reviewer #1: Yes

Reviewer #4: Yes

**********

5. Is the manuscript presented in an intelligible fashion and written in standard English?

PLOS ONE does not copyedit accepted manuscripts, so the language in submitted articles must be clear, correct, and unambiguous. Any typographical or grammatical errors should be corrected at revision, so please note any specific errors here.

Reviewer #1: Yes

Reviewer #4: Yes

**********

6. Review Comments to the Author

Please use the space provided to explain your answers to the questions above. You may also include additional comments for the author, including concerns about dual publication, research ethics, or publication ethics. (Please upload your review as an attachment if it exceeds 20,000 characters)

Reviewer #1: Authors had satisfactorily addressed all issues raised by reviewer and now ready for acceptance. It is ready for next phase of editorial process.

Reviewer #4: Thank you for the opportunity to review this paper. It’s a strong study that focuses on a crucial health issue and an important target group. However, I have a few comments for the authors to consider:

1. In the quantitative phase, please clarify and justify why only 30 participants were selected, especially given that many were eligible.

2. Explain why systematic random sampling was chosen for participant selection in the quantitative phase.

3. With such a small sample size, are you confident that a Chi-square test is appropriate? It might be worth considering Fisher’s exact test depending on the data.

4. For the qualitative phase, please clarify the language criteria for participants and the interview process. How were interviews conducted, and what were the interviewers’ language proficiencies? If non-English languages were used, describe the translation and transcription processes.

5. Regarding study rigor and trustworthiness in the qualitative phase, please detail how you addressed criteria like transferability, credibility, dependability, and confirmability.

**********

7. PLOS authors have the option to publish the peer review history of their article (what does this mean?). If published, this will include your full peer review and any attached files.

If you choose “no”, your identity will remain anonymous but your review may still be made public.

**Do you want your identity to be public for this peer review?**   For information about this choice, including consent withdrawal, please see our Privacy Policy.

Reviewer #1: No

Reviewer #4: **Yes:**  Masoud Mohammadnezhad

**********

[NOTE: If reviewer comments were submitted as an attachment file, they will be attached to this email and accessible via the submission site. Please log into your account, locate the manuscript record, and check for the action link “View Attachments”. If this link does not appear, there are no attachment files.]

To ensure your figures meet our technical requirements, please review our figure guidelines: https://journals.plos.org/plosone/s/figures

You may also use PLOS’s free figure tool, NAAS, to help you prepare publication quality figures: https://journals.plos.org/plosone/s/figures#loc-tools-for-figure-preparation.

NAAS will assess whether your figures meet our technical requirements by comparing each figure against our figure specifications.

# Author comment: Author response to Decision Letter 2

**DOI:** 10.1371/journal.pone.0332565.r004

17 Feb 2026

February 15, 2026

Maheshkumar Baladaniya, PT, MS, DPT

Academic Editor, PLOS ONE

Response to Reviewers

RE: “You never know what’s in front of you”: A mixed methods study of barriers and facilitators to physical activity among blind and low vision adults with type 2 diabetes (Submission ID: PONE-D-25-47274)

Dear Dr. Baladaniya,

Thank you for the opportunity to revise our manuscript. We have carefully revised the paper in response to the reviewers’ recommendations. We are grateful to you and the reviewers for the thoughtful, constructive feedback throughout the review process, resulting in a strengthened manuscript.

In the response below, we address each reviewer’s comment and indicate the corresponding line numbers where revisions can be found in the revised, unmarked version of the manuscript. All substantive edits are also visible in the marked-up version of the manuscript (“Revised Manuscript with Track Changes”), in accordance with editorial guidelines.

We appreciate the time and expertise invested by the editorial team and reviewers, and we thank you for considering this revised manuscript for publication in PLOS ONE.

Sincerely,

Emily J. Nicklett, PhD, MSW (Corresponding Author)

Associate Professor of Social Work and Public Health

College for Health, Community and Policy

University of Texas at San Antonio

Email: Emily.Nicklett@utsa.edu

Editorial Comments:

1. If the reviewer comments include a recommendation to cite specific previously published works, please review and evaluate these publications to determine whether they are relevant and should be cited. There is no requirement to cite these works unless the editor has indicated otherwise.

We appreciate this guidance. No specific citations were recommended by the reviewers in this round of review.

2. Please review your reference list to ensure that it is complete and correct. If you have cited papers that have been retracted, please include the rationale for doing so in the manuscript text, or remove these references and replace them with relevant current references. Any changes to the reference list should be mentioned in the rebuttal letter that accompanies your revised manuscript. If you need to cite a retracted article, indicate the article’s retracted status in the References list and also include a citation and full reference for the retraction notice.

We carefully reviewed our reference list to ensure completeness and accuracy and corrected minor errors. None of the cited papers have been retracted. One article previously cited as a preprint (Lee et al., 2026) has since been formally published, and the final publication details have now been updated in the reference list. In addition, we added one new reference (Lincoln & Guba, 1985) in response to reviewer feedback (Reviewer 4, Comment 5), as discussed below.

Reviewer 1:

1. All comments have been addressed. Authors had satisfactorily addressed all issues raised by the reviewer and are now ready for acceptance. It is ready for the next phase of the editorial process.

We appreciate the feedback and find that the manuscript has been significantly strengthened due to the reviewer’s suggestions.

Reviewer 4:

1. In the quantitative phase, please clarify and justify why only 30 participants were selected, especially given that many were eligible.

Thank you for this comment. We have clarified this point in the Participant Recruitment section (lines 123-155). Although a larger number of individuals met eligibility criteria, enrollment was intentionally limited to 30 participants to align with the exploratory objectives of the quantitative phase and to ensure feasibility within the study’s resource and accessibility constraints. The quantitative component was designed to provide descriptive characteristics of the sample, characterize preliminary patterns, and support integration with qualitative findings rather than to serve as a fully powered hypothesis-testing analysis. The manuscript has been revised to reflect this rationale and to ensure appropriate interpretation of the quantitative results (lines 124-143; 983-987).

2. Explain why systematic random sampling was chosen for participant selection in the quantitative phase.

Thank you for this comment. We have clarified the sampling approach in the manuscript (line 139). Participant selection for the quantitative phase used simple random sampling, not systematic random sampling. Eligible clients were randomly selected from the organizational roster using a computer-generated random number sequence so that all eligible individuals had an equal probability of selection. The methods section has been revised to reflect this more precise terminology and to briefly explain the rationale for this approach (lines 136-147; Fig 1).

3. With such a small sample size, are you confident that a Chi-square test is appropriate? It might be worth considering Fisher’s exact test depending on the data.

We appreciate this suggestion. Given our small sample (N=30) and the presence of sparse/zero cell counts in several categorical predictors, we re-estimated all categorical associations using Fisher’s exact tests. Results and conclusions remained unchanged. Because Fisher’s exact test provides valid inferences for sparse contingency tables, we have updated the Methods (lines 242-247), Results (lines 282-287), Table 3 (line 288), and table footnotes (lines 291-294) to reflect this revision.

4. For the qualitative phase, please clarify the language criteria for participants and the interview process. How were interviews conducted, and what were the interviewers’ language proficiencies? If non-English languages were used, describe the translation and transcription processes.

Thank you for this comment. We have clarified the language criteria and interview procedures in the Methods section (lines 163-169). Participants were eligible to complete interviews in either English or Spanish, according to their language preference. All interviews were conducted by trained members of the study team; Spanish-language interviews were conducted by bilingual (English-Spanish) interviewers. The qualitative interview guide and quantitative instruments were translated into Spanish and verified by two bilingual members of the study team before data collection.

Interviews conducted in English and Spanish followed the same semi-structured protocol and were administered in a comparable manner. All interviews were audio-recorded, transcribed verbatim, and, for Spanish-language interviews, translated into English by two bilingual interviewers (trained in qualitative methods) who conducted the interviews. This approach ensured conceptual consistency across languages and minimized loss of meaning during translation. The manuscript has been revised to reflect these procedures (lines 157-169).

5. Regarding study rigor and trustworthiness in the qualitative phase, please detail how you addressed criteria like transferability, credibility, dependability, and confirmability.

Thank you for this comment. Drawing on Lincoln & Guba (1985), we have clarified procedures used to enhance rigor and trustworthiness in the qualitative phase, guided by established qualitative criteria (e.g., transferability, credibility, dependability, and confirmability).

Credibility was enhanced through co-development of the interview guide with the community partner, semi-structured interviews, investigator triangulation, piloting before implementation with members of the target population, and collaborative analysis with the community partner. Two researchers independently coded line-by-line coding of all transcripts and met regularly to discuss interpretations, resolve discrepancies, and reach consensus. Engagement with the community partner throughout the study design, data collection, and interpretation further strengthened credibility.

Dependability was addressed by using a systematic, multi-stage analytic process, detailed analytic memoing, and a maintained audit trail of analytic decisions. Coding decisions, analytic memos, and iterative refinements to the codebook were documented, enabling consistency in analytic procedures across transcripts.

Confirmability was enhanced through reflexive practices, double-coding, and carefully grounding interpretations in verbatim participant quotations. Researchers engaged in reflexive discussions to examine assumptions and potential biases, and analytic decisions were grounded in participants’ verbatim data. The use of double-coding and consensus meetings further reduced individual researcher bias.

Transferability was supported by providing rich, thick descriptions of the study context, participant characteristics, and analysis procedures, allowing readers to assess the applicability of findings to other settings or populations. While the study is not intended to produce statistically generalizable findings, detailed contextual information enables analytic transferability.

We incorporated an explicit discussion of these strategies in the Methods section (lines 223-232). We appreciate this important feedback, which has helped us enhance the rigor and transparency of the qualitative component.

Attachment Submitted filename: Response to Reviewers r2 2.15.26.docx

# Peer review: Decision Letter 2

**DOI:** 10.1371/journal.pone.0332565.r005

**Authors:** Maheshkumar Baladaniya

12 Apr 2026

PONE-D-25-47274R2

“You never know what’s in front of you”: A mixed methods study of barriers and facilitators to physical activity among blind and low-vision adults with type 2 diabetes

PLOS One

Dear Dr. Nicklett,

Thank you for submitting your manuscript to PLOS ONE. After careful consideration, we feel that it has merit but does not fully meet PLOS ONE’s publication criteria as it currently stands. Therefore, we invite you to submit a revised version of the manuscript that addresses the points raised during the review process.

Please submit your revised manuscript by May 27 2026 11:59PM. If you will need more time than this to complete your revisions, please reply to this message or contact the journal office at plosone@plos.org. When you're ready to submit your revision, log on to https://www.editorialmanager.com/pone/ and select the 'Submissions Needing Revision' folder to locate your manuscript file.

Please include the following items when submitting your revised manuscript:

- A letter that responds to each point raised by the academic editor and reviewer(s). You should upload this letter as a separate file labeled 'Response to Reviewers'.
- A marked-up copy of your manuscript that highlights changes made to the original version. You should upload this as a separate file labeled 'Revised Manuscript with Track Changes'.
- An unmarked version of your revised paper without tracked changes. You should upload this as a separate file labeled 'Manuscript'.

If you would like to make changes to your financial disclosure, please include your updated statement in your cover letter. Guidelines for resubmitting your figure files are available below the reviewer comments at the end of this letter.

If applicable, we recommend that you deposit your laboratory protocols in protocols.io to enhance the reproducibility of your results. Protocols.io assigns your protocol its own identifier (DOI) so that it can be cited independently in the future. For instructions see: https://journals.plos.org/plosone/s/submission-guidelines#loc-laboratory-protocols. Additionally, PLOS ONE offers an option for publishing peer-reviewed Lab Protocol articles, which describe protocols hosted on protocols.io. Read more information on sharing protocols at https://plos.org/protocols?utm_medium=editorial-email&utm_source=authorletters&utm_campaign=protocols.

As the corresponding author, your ORCID iD is verified in the submission system and will appear in the published article. PLOS supports the use of ORCID, and we encourage all coauthors to register for an ORCID iD and use it as well. Please encourage your coauthors to verify their ORCID iD within the submission system before final acceptance, as unverified ORCID iDs will not appear in the published article. *Only*   the individual author can complete the verification step; PLOS staff *cannot*   verify ORCID iDs on behalf of authors.

We look forward to receiving your revised manuscript.

Kind regards,

Maheshkumar Baladaniya

Academic Editor

PLOS One

Journal Requirements:

If the reviewer comments include a recommendation to cite specific previously published works, please review and evaluate these publications to determine whether they are relevant and should be cited. There is no requirement to cite these works unless the editor has indicated otherwise.

Please review your reference list to ensure that it is complete and correct. If you have cited papers that have been retracted, please include the rationale for doing so in the manuscript text, or remove these references and replace them with relevant current references. Any changes to the reference list should be mentioned in the rebuttal letter that accompanies your revised manuscript. If you need to cite a retracted article, indicate the article’s retracted status in the References list and also include a citation and full reference for the retraction notice.

Additional Editor Comments:

Minor changes required as reviewer has suggested adn raised the questions.

[Note: HTML markup is below. Please do not edit.]

Reviewers' comments:

Reviewer's Responses to Questions

**Comments to the Author**

1. If the authors have adequately addressed your comments raised in a previous round of review and you feel that this manuscript is now acceptable for publication, you may indicate that here to bypass the “Comments to the Author” section, enter your conflict of interest statement in the “Confidential to Editor” section, and submit your “Accept” recommendation.

Reviewer #5: All comments have been addressed

Reviewer #6: All comments have been addressed

Reviewer #7: All comments have been addressed

**********

2. Is the manuscript technically sound, and do the data support the conclusions?

The manuscript must describe a technically sound piece of scientific research with data that supports the conclusions. Experiments must have been conducted rigorously, with appropriate controls, replication, and sample sizes. The conclusions must be drawn appropriately based on the data presented.

Reviewer #5: Yes

Reviewer #6: Yes

Reviewer #7: Yes

**********

3. Has the statistical analysis been performed appropriately and rigorously?

Reviewer #5: Yes

Reviewer #6: Yes

Reviewer #7: No

**********

4. Have the authors made all data underlying the findings in their manuscript fully available?

The PLOS Data policy requires authors to make all data underlying the findings described in their manuscript fully available without restriction, with rare exception (please refer to the Data Availability Statement in the manuscript PDF file). The data should be provided as part of the manuscript or its supporting information, or deposited to a public repository. For example, in addition to summary statistics, the data points behind means, medians and variance measures should be available. If there are restrictions on publicly sharing data—e.g. participant privacy or use of data from a third party—those must be specified.

Reviewer #5: Yes

Reviewer #6: Yes

Reviewer #7: Yes

**********

5. Is the manuscript presented in an intelligible fashion and written in standard English?

PLOS ONE does not copyedit accepted manuscripts, so the language in submitted articles must be clear, correct, and unambiguous. Any typographical or grammatical errors should be corrected at revision, so please note any specific errors here.

Reviewer #5: Yes

Reviewer #6: Yes

Reviewer #7: Yes

**********

6. Review Comments to the Author

Please use the space provided to explain your answers to the questions above. You may also include additional comments for the author, including concerns about dual publication, research ethics, or publication ethics. (Please upload your review as an attachment if it exceeds 20,000 characters)

Reviewer #5: 148 - your recruitment methods seem expertly done and the demographics of subjects is good

170 - the close-ended questionnaires appear appropriate and ADI, Walk Score, Transit Score all seem valid.

223 - the data processing and cross checking methods appear robust

Table 4 - this is very helpful and the themes/sub-themes are well reasoned

Fig 4 - well organized and useful recommendations for the intended audience of this work

Reviewer #6: This is a meaningful mixed-methods study on physical activity among adults with type 2 diabetes and blindness/low vision. The manuscript has improved substantially through revision, particularly by clarifying recruitment procedures, interview protocols, qualitative rigor, and the overall presentation of the mixed-methods design.

My main remaining concern is the statistical presentation in Table 3. The manuscript states that Fisher’s exact tests were used for categorical predictors because of small cell counts, yet single odds ratios are reported for multi-category variables such as age group and employment status. It is unclear how these ORs were derived, and the current presentation may confuse readers. The authors should either report only omnibus Fisher’s exact p-values or reanalyze these variables using clearly specified logistic regression models with reference categories and category-specific ORs with 95% CIs.

A second issue is the wording around sample size. The manuscript describes the quantitative component as exploratory but also suggests that the sample size was adequate for adequately powered bivariate comparisons, while the Discussion later acknowledges limited power. This language should be made more consistent.

Reviewer #7: (No Response)

**********

7. PLOS authors have the option to publish the peer review history of their article (what does this mean?). If published, this will include your full peer review and any attached files.

If you choose “no”, your identity will remain anonymous but your review may still be made public.

**Do you want your identity to be public for this peer review?**   For information about this choice, including consent withdrawal, please see our Privacy Policy.

Reviewer #5: No

Reviewer #6: No

Reviewer #7: No

**********

[NOTE: If reviewer comments were submitted as an attachment file, they will be attached to this email and accessible via the submission site. Please log into your account, locate the manuscript record, and check for the action link “View Attachments”. If this link does not appear, there are no attachment files.]

To ensure your figures meet our technical requirements, please review our figure guidelines: https://journals.plos.org/plosone/s/figures

You may also use PLOS’s free figure tool, NAAS, to help you prepare publication quality figures: https://journals.plos.org/plosone/s/figures#loc-tools-for-figure-preparation.

NAAS will assess whether your figures meet our technical requirements by comparing each figure against our figure specifications.

# Author comment: Author response to Decision Letter 3

**DOI:** 10.1371/journal.pone.0332565.r006

20 Apr 2026

April 20, 2026

Maheshkumar Baladaniya, PT, MS, DPT

Academic Editor, PLOS ONE

Response to Reviewers

RE: “You never know what’s in front of you”: A mixed methods study of barriers and facilitators to physical activity among blind and low vision adults with type 2 diabetes (Submission ID: PONE-D-25-47274)

Dear Dr. Baladaniya,

Thank you for the opportunity to revise our manuscript. We have carefully revised the paper in response to the reviewers’ recommendations. We are grateful for the constructive feedback, which has resulted in a more cohesive and methodologically transparent manuscript.

Key revisions in this version include:

- Statistical Harmonization: We now employ Cramér’s V effect sizes, a more mathematically appropriate measure of association, without the need for reference-group modeling.

- Framing and Consistency: We have removed conflicting claims regarding sample size “adequacy”. The manuscript now consistently frames N=30 as a limitation, while justifying the exploratory use of exact statistical methods. We ensured that the variables shown in Tables 2 and 3 consistently followed sentence case.

- Limitations: The discussion section has been expanded to explicitly acknowledge the trade-offs of our statistical choices, including the conservative nature of Fisher’s exact test and the sensitivity of omnibus effect sizes to table dimensions.

Below, we address the Reviewer comments and feedback, indicating the corresponding line numbers where revisions can be found in the unmarked version of the manuscript. All substantive edits are also visible in the marked-up version of the manuscript.

We appreciate the time and expertise invested by the editorial team and reviewers, and we thank you for considering this revised manuscript for publication in PLOS ONE.

Sincerely,

Emily J. Nicklett, PhD, MSW (Corresponding Author)

Associate Professor of Social Work and Public Health

College for Health, Community and Policy

University of Texas at San Antonio

Email: Emily.Nicklett@utsa.edu

------------------------

Editorial Comments:

1. If the reviewer comments include a recommendation to cite specific previously published works, please review and evaluate these publications to determine whether they are relevant and should be cited. There is no requirement to cite these works unless the editor has indicated otherwise.

We appreciate this guidance. No specific citations were recommended by the reviewers in this round of review.

2. Please review your reference list to ensure that it is complete and correct. If you have cited papers that have been retracted, please include the rationale for doing so in the manuscript text, or remove these references and replace them with relevant current references. Any changes to the reference list should be mentioned in the rebuttal letter that accompanies your revised manuscript. If you need to cite a retracted article, indicate the article’s retracted status in the References list and also include a citation and full reference for the retraction notice.

We carefully reviewed our reference list to ensure completeness and accuracy.

------------------------

Reviewer 5:

1. Your recruitment methods seem expertly done, and the demographics of the subjects is good. The closed-ended questionnaires appear appropriate, and ADI, Walk Score, and Transit Score all seem valid. The data processing and cross-checking methods appear robust. Table 4 is very helpful, and the theme/sub-themes are well-reasoned. Figure 4 is well-organized and provides useful recommendations for the intended audience of this work.

We appreciate the positive feedback.

------------------------

Reviewer 6:

1. This is a meaningful mixed-methods study on physical activity among adults with type 2 diabetes and blindness/low vision. The manuscript has improved substantially through revision, particularly by clarifying recruitment procedures, interview protocols, qualitative rigor, and the overall presentation of the mixed-method design.

We are grateful for these comments and agree that the manuscript has greatly improved through the revision process.

2. My remaining concern is the statistical presentation in Table 3. The manuscript states that Fisher’s exact tests were used for categorical predictors of small cell counts, yet single odds ratios are reported for multi-category variables such as age groups and employment status. It is unclear how these ORs were derived, and the current presentation may confuse readers. The reporters should either report only omnibus Fisher’s exact p-values or reanalyze these variables using clearly specified logistic regression models with reference categories and category-specific ORs with 95% CIs.

We thank the Reviewers for this insightful comment regarding the ambiguity of reporting a single Odds Ratio for multi-category variables (e.g., age group, race/ethnicity). We agree that a single OR in these instances lacks a clear reference point and can be mathematically misleading.

To resolve this and ensure statistical clarity, we have updated our presentation of effect sizes in Table 3 as follows:

a) For multicategory variables (>2 levels), we have replaced the single OR with Cramér’s V. This provides an appropriate omnibus measure of association that is not dependent on a reference category.

b) For binary and continuous variables, we have retained the Odds Ratio (OR) as the measure of association, as it remains the standard and most interpretable effect size for these data types.

c) Justification for Approach: While the Reviewer suggested Category-specific ORs via logistic regression, we opted for the omnibus Cramér’s V approach given our small sample size (N=30), as reference-group modeling with small cell counts can result in unstable estimates and “not estimable” parameters (as seen in some of our descriptive cells), whereas Cramér’s V remains a robust and informative indicator of effect magnitude (Agresti, 2013).

These changes are now reflected in the Methods section (lines 242-250), Table 3 and its associated Note (lines 292-299), and the updated Results for the bivariate analysis (lines 285-291), and the Discussion section (lines 988-998).

3. A second issue is the wording around sample size. The manuscript describes the quantitative component as exploratory but also suggests that the sample size was adequate for bivariate comparisons, while the Discussion later acknowledges limited power. The language should be made more consistent.

We appreciate the Reviewer’s call for consistency regarding our sample size description. We have revised the manuscript to remove conflicting claims of “adequacy” while maintaining that the analyses are exploratory. We now consistently frame the N = 30 sample as a limitation that necessitated the use of exact statistical methods (Fisher’s exact tests) and omnibus effect sizes (Cramér’s V), which are methodologically appropriate for small-cell bivariate associations even when overall statistical power is limited. Additionally, we have expanded the Limitations section to explicitly acknowledge the inherent trade-offs of these methods, including the conservative nature of Fisher’s exact test and the sensitivity of Cramér’s V to table dimensions and empty cells. These changes are now reflected in the Methods (lines 136-137) and Discussion (lines 988-998) sections.

------------------------

Reviewer 7:

N/A – All comments have been addressed.

We appreciate the positive feedback.

------------------------

We are grateful to the anonymous Reviewers for their constructive feedback and insights.

Attachment Submitted filename: Response to Reviewers r3.docx

# Peer review: Decision Letter 3

**DOI:** 10.1371/journal.pone.0332565.r007

**Authors:** Taiwo Opeyemi Aremu

2 Jun 2026

“You never know what’s in front of you”: A mixed methods study of barriers and facilitators to physical activity among blind and low-vision adults with type 2 diabetes

PONE-D-25-47274R3

Dear Dr. Nicklett,

We’re pleased to inform you that your manuscript has been judged scientifically suitable for publication and will be formally accepted for publication once it meets all outstanding technical requirements.

Within one week, you’ll receive an e-mail detailing the required amendments. When these have been addressed, you’ll receive a formal acceptance letter and your manuscript will be scheduled for publication.

An invoice will be generated when your article is formally accepted. Please note, if your institution has a publishing partnership with PLOS and your article meets the relevant criteria, all or part of your publication costs will be covered. Please make sure your user information is up-to-date by logging into Editorial Manager at Editorial Manager® and clicking the ‘Update My Information' link at the top of the page. For questions related to billing, please contact billing support.

If your institution or institutions have a press office, please notify them about your upcoming paper to help maximize its impact. If they’ll be preparing press materials, please inform our press team as soon as possible -- no later than 48 hours after receiving the formal acceptance. Your manuscript will remain under strict press embargo until 2 pm Eastern Time on the date of publication. For more information, please contact onepress@plos.org.

Kind regards,

Taiwo Opeyemi Aremu, MD, MPH, PhD

Academic Editor

PLOS One

Additional Editor Comments (optional):

Reviewers' comments:

Reviewer's Responses to Questions

**Comments to the Author**

1. If the authors have adequately addressed your comments raised in a previous round of review and you feel that this manuscript is now acceptable for publication, you may indicate that here to bypass the “Comments to the Author” section, enter your conflict of interest statement in the “Confidential to Editor” section, and submit your “Accept” recommendation.

Reviewer #5: (No Response)

Reviewer #6: All comments have been addressed

**********

2. Is the manuscript technically sound, and do the data support the conclusions?

The manuscript must describe a technically sound piece of scientific research with data that supports the conclusions. Experiments must have been conducted rigorously, with appropriate controls, replication, and sample sizes. The conclusions must be drawn appropriately based on the data presented.

Reviewer #5: (No Response)

Reviewer #6: Yes

**********

3. Has the statistical analysis been performed appropriately and rigorously?

Reviewer #5: (No Response)

Reviewer #6: Yes

**********

4. Have the authors made all data underlying the findings in their manuscript fully available?

The PLOS Data policy requires authors to make all data underlying the findings described in their manuscript fully available without restriction, with rare exception (please refer to the Data Availability Statement in the manuscript PDF file). The data should be provided as part of the manuscript or its supporting information, or deposited to a public repository. For example, in addition to summary statistics, the data points behind means, medians and variance measures should be available. If there are restrictions on publicly sharing data—e.g. participant privacy or use of data from a third party—those must be specified.

Reviewer #5: (No Response)

Reviewer #6: Yes

**********

5. Is the manuscript presented in an intelligible fashion and written in standard English?

PLOS ONE does not copyedit accepted manuscripts, so the language in submitted articles must be clear, correct, and unambiguous. Any typographical or grammatical errors should be corrected at revision, so please note any specific errors here.

Reviewer #5: (No Response)

Reviewer #6: Yes

**********

6. Review Comments to the Author

Please use the space provided to explain your answers to the questions above. You may also include additional comments for the author, including concerns about dual publication, research ethics, or publication ethics. (Please upload your review as an attachment if it exceeds 20,000 characters)

Reviewer #5: (No Response)

Reviewer #6: (No Response)

**********

7. PLOS authors have the option to publish the peer review history of their article (what does this mean?). If published, this will include your full peer review and any attached files.

If you choose “no”, your identity will remain anonymous but your review may still be made public.

**Do you want your identity to be public for this peer review?**   For information about this choice, including consent withdrawal, please see our Privacy Policy.

Reviewer #5: **Yes:**  Gregory Hopkins, OD MS

Reviewer #6: No

**********

# Editor report: Acceptance letter

**DOI:** 10.1371/journal.pone.0332565.r008

**Authors:** Taiwo Opeyemi Aremu

PONE-D-25-47274R3

PLOS One

Dear Dr. Nicklett,

I'm pleased to inform you that your manuscript has been deemed suitable for publication in PLOS One. Congratulations! Your manuscript is now being handed over to our production team.

At this stage, our production department will prepare your paper for publication. This includes ensuring the following:

* All references, tables, and figures are properly cited

* All relevant supporting information is included in the manuscript submission,

* There are no issues that prevent the paper from being properly typeset

You will receive further instructions from the production team, including instructions on how to review your proof when it is ready. Please keep in mind that we are working through a large volume of accepted articles, so please give us a few days to review your paper and let you know the next and final steps.

Lastly, if your institution or institutions have a press office, please let them know about your upcoming paper now to help maximize its impact. If they'll be preparing press materials, please inform our press team within the next 48 hours. Your manuscript will remain under strict press embargo until 2 pm Eastern Time on the date of publication. For more information, please contact onepress@plos.org.

You will receive an invoice from PLOS for your publication fee after your manuscript has reached the completed accept phase. If you receive an email requesting payment before acceptance or for any other service, this may be a phishing scheme. Learn how to identify phishing emails and protect your accounts at https://explore.plos.org/phishing.

If we can help with anything else, please email us at customercare@plos.org.

Thank you for submitting your work to PLOS ONE and supporting open access.

Kind regards,

PLOS ONE Editorial Office Staff

on behalf of

Dr. Taiwo Opeyemi Aremu

Academic Editor

PLOS One
